# Lobophytones O–T, New Biscembranoids and Cembranoid from Soft Coral *Lobophytum pauciflorum*

**DOI:** 10.3390/md8112848

**Published:** 2010-11-10

**Authors:** Pengcheng Yan, Zhiwei Deng, Leen van Ofwegen, Peter Proksch, Wenhan Lin

**Affiliations:** 1 State Key Laboratory of Natural and Biomimetic Drugs, Peking University, Beijing 100083, China; E-Mail: ypc@bjmu.edu.cn; 2 Analytical and Testing Center, Beijing Normal University, Beijing, 100875, China; E-Mail: dengzw@bnu.edu.cn; 3 Netherlands Centre for Biodiversity Naturalis, 2300 RA Leiden, The Netherlands; E-Mail: leen.vanofwegen@ncbnaturalis.nl; 4 Institute of Pharmaceutical Biology and Biotechnology, Heinrich-Heine University, 40225 Duesseldorf, Germany; E-Mail: proksch@duesseldorf-uni.de

**Keywords:** soft coral, *Lobophytum pauciflorum*, lobophytones O-T, structure elucidation, NO inhibition, antibiotic activity

## Abstract

Chemical examination of a Chinese soft coral *Lobophytum pauciflorum* resulted in the isolation of five new biscembranoids named lobophytones O–S (**1**–**5**) and a new “monomeric” cembrane lobophytone T (**6**). The structures of the new compounds were elucidated by interpretation of 1D and 2D NMR (COSY, HSQC, HMBC, and NOESY) spectroscopic data in association with MS and IR data. Lobophytone Q showed significant inhibition against lipopolysaccharide (LPS)-induced nitric oxide (NO) release in mouse peritoneal macrophages, while lobophytones Q and T showed inhibitory activities against the bacteria *S. aureus* and *S. pneumoniae.*

## 1. Introduction

**Figure 1:**
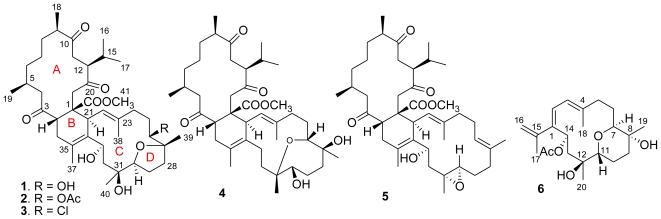
Structures of lobophytones O–T (**1**–**6**).

Biscembranes are a class of marine natural products with unusual structure patterns, which are mainly found in the marine soft coral genus of *Sarcophyton* (*S. tortuosum*, *S. glaucum*, *S. latum*, and *S. elegans*). They are supposed to be biogenetically derived by a Diels–Alder cycloaddition of cembranoid-diene and cembranoid-dienophile [1–8]. Recently, we found the soft coral *Lobophytum pauciflorum* also contains a variety of biscembrane analogues, but they presented the structure pattern with antipodal Diels–Alder cycloaddition and were named isobiscembranoids [9]. This finding provided a new marine organism to enrich the chemical diversity of biscembranoids. Our continuing interest in the chemical diversity of this specimen led to the isolation of five new biscembranoids (**1**–**5**) and a new monomeric cembrane (**6**) (Figure 1). This paper reports the structural elucidation of these new compounds and their bioactivity.

## 2. Results and Discussion

Repeated column chromatography of the EtOAc-soluble fraction obtained from the EtOH extract of the soft coral *L. pauciflorum* led to the isolation of five biscembranoids and a cembranoid. All biscembranoids shared the partial structure related to methyl tortuosoate, a cembranoid-dienophile [6].

Lobophytone O (**1**) has a molecular formula of C_41_H_64_O_9_ as determined by HR-ESIMS *m/z* 723.4428 [M + Na]^+^ (calcd for C_41_H_64_NO_9_Na, 723.4442) and NMR data, requiring 10 degrees of unsaturation. The ^1^H NMR spectrum exhibited the resonances for nine methyls including two olefinic methyl singlets (*δ*_H_ 1.65, 1.66) and an OMe (*δ*_H_ 3.42), while the ^13^C NMR spectrum presented 41 carbon resonances including three ketone and one ester carbonyl carbon, four olefinic carbons, and five oxygen-bearing *sp*^3^ carbons. Six degrees of unsaturation, as accounted by the functional groups out of 10 in the molecule, suggested the presence of a tetracyclic nucleus. The ^1^H and ^13^C NMR data of **1** featured a biscembrane skeleton, closely related to that of ximaolide C as formerly isolated from *Sarcophyton tortuosum* [3]. COSY in association with HMQC relationships enabled all protons and their corresponding carbons in the molecule to be assigned (Tables 1 and 2). Comparison of NMR data revealed that rings A and B of **1** were the same as in ximaolide C. The difference was found in ring C, where a hydroxy group of **1** replaced a chlorine atom at C-31 of ximaolide C. This was evident from the presence of a hydroxy proton at *δ*_H_ 3.98 (s), and its HMBC correlations with C-30 (*δ*_C_ 89.2) and C-31 (*δ*_C_ 73.6), and by the missing chlorine isotope pattern in the mass spectrum.

**Figure 2:**
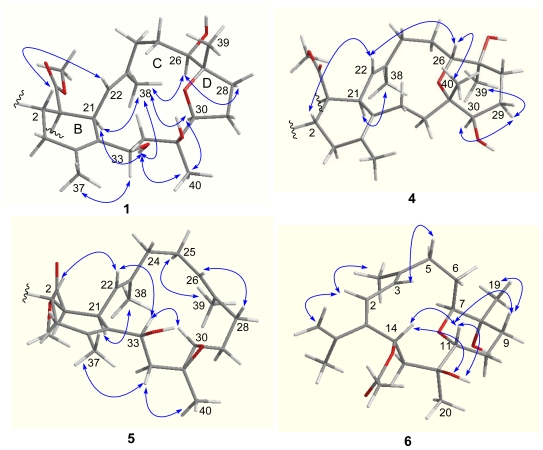
Key NOE correlations of **1**, **4**–**6** related to rings B–D.

The relative configurations of the stereogenic centers in **1** were determined on the basis of NOE relationships (Figure 2) and *J* values. The NOE relationship between H-2 (*δ*_H_ 3.47) and OMe suggested *cis*-conjunction of rings A and B. The exclusive *β*-face of the methyl ester group as found in all known biscembranes led to the biogenetical assignment of the methyl ester at C-1 of **1** to be *β*-oriented. The obvious NOE correlation between H-22 (*δ*_H_ 4.78)/H-2 suggested the spatial vicinity of the two protons, thus H-21 was *α*-oriented. The NOE correlations between H-21 (*δ*_H_ 3.38)/H_3_-38 (*δ*_H_ 1.65), H_3_-38/H-26 (*δ*_H_ 3.01), and H_3_-38/H-30 (*δ*_H_ 3.77) indicated α-orientations of H-26 and H-30, as well as 22*E* configuration. The β-orientation of H_3_-39 (*δ*_H_ 0.99) was evident from the NOE relationship between H-26 and H-28a (*δ*_H_ 2.25), which further correlated to H-30, while the assignment of H-33β was based on the NOE interactions from OH-33 (*δ*_H_ 4.40) to H-21, H_3_-38, and H_3_-40 (Figure 2). In addition, the NOE relationship between H-30/H_3_-40 (*δ*_H_ 0.97) suggested H_3_-40 to be *α*-oriented.

Lobophytone P (**2**) has a molecular formula of C_43_H_66_O_10_ as determined by HR-ESIMS (*m*/*z* 765.4570 [M + Na]^+^), indicating 42 amu more than that of **1**. Comparison of NMR data revealed that the structure of **2** was closely related to that of **1**, except for the presence of an acetyl group as evident from the ^13^C NMR signals at *δ*_C_ 171.3 (Cq) and 21.4 (CH_3_) and the corresponding ^1^H NMR signal at *δ*_H_ 2.06 (3H, s). The HMBC correlation from H-26 (*δ*_H_ 4.56, d, *J* = 10.8 Hz) to the acetyl carbonyl carbon ascertained the location of the acetoxy group at C-26. The similar NOE and NMR data of **2** and **1** indicated that **2** shared the same configurations of the stereogenic centers of **1**. Thus, compound **2** was determined to be a C-26 acetylated analogue of **1**.

Lobophytone Q (**3**) has a molecular formula of C_41_H_63_O_8_Cl as determined by HR-ESIMS (*m/z* 741.4096 [M + Na]^+^, calcd 741.4104), indicating 10 degrees of unsaturation and the presence of a chlorine atom. The 1D and 2D NMR spectroscopic analysis revealed that the structure of **3** was also closely related to that of **1**. The presence of two D_2_O exchangeable protons at *δ*_H_ 4.18 (s) and 4.46 (br) was attributed to two hydroxy groups, which were deduced to be attached at C-31 and C-33, based on the HMBC interactions from OH-31 (*δ*_H_ 4.18, s) to C-30 (*δ*_C_ 90.1), C-31 (*δ*_C_ 73.2) and C-32 (*δ*_C_ 40.7), and the COSY correlation between OH-33 (*δ*_H_ 4.46, bs) and H-33 (*δ*_H_ 4.82). The chlorine atom of **3** was assumed to be substituted at C-26 according to the upfield-shifted C-26 (*δ*_C_ 65.1), which showed around Δδ 5 less than the same carbon of **1** linked by hydroxy group. The relative configurations of the chiral centers in **3** were in agreement with those of **1** based on the similar NMR and NOE data.

The molecular formula of lobophytone R (**4**) was determined as C_41_H_64_O_8_ on the basis of HR-ESIMS (*m*/*z* 685.4670 [M + H]^+^), implying 10 degrees of unsaturation. Comparison of NMR data indicated that **4** is a biscembranoid possessing the same partial structure, with respect to rings A and B, of **1**. However, the obvious downfield shifted C-26 (*δ*_C_ 77.8) and C-31 (*δ*_C_ 80.5), and the upfield shifted C-27 (*δ*_C_ 74.7) and C-30 (*δ*_C_ 80.0), in comparison with the corresponding carbons of **1**, suggested that **4** contains an ether bridge at C-26/C-31 in ring C rather than C-27/C-30 ether bond of **1**. The HMBC correlation from H-26 (*δ*_H_ 3.39) to C-31 confirmed this assignment. The COSY correlation between OH (*δ*_H_ 4.49, br)/H-30 (*δ*_H_ 2.91) and the HMBC relationships from OH (*δ*_H_ 4.18) to C-26 and C-27 disclosed two hydroxy groups at C-27 and C-30, respectively. The NOE relationships observed between H-2 (*δ*_H_ 3.49, m)/H-22 (*δ*_H_ 4.94, d, *J* = 11.5 Hz), H-22/H-26, and H-26/H_3_-40 (*δ*_H_ 0.92) indicated H-26 and H_3_-40 to be *β*-oriented, while the NOE correlations between OH-27 (*δ*_H_ 4.18)/H-26, H-30 (*δ*_H_ 2.91, m)/H-29a (*δ*_H_ 1.31, m), and H_3_-39 (*δ*_H_ 0.98)/H-29a assigned to H_3_-39α and H-30α.

Lobophytone S (**5**) has a molecular formula of C_41_H_62_O_7_, as determined by HR-ESIMS (*m*/*z* 689.4384 [M + Na]^+^). The NMR spectroscopic data of **5** (Tables 1 and 2) indicated that it is a biscembrane, structurally related to ximaolide A [3], a biscembrane isolated from soft coral *Sarcophyton tortuosum*. Analysis of the NMR spectroscopic data revealed an additional vinyl group (*δ*_C_ 125.1, 132.8) in ring C of **5**, in addition to the double bond located at C-22/C-23. This double bond was deduced to be located at C-26/C-27 based on the COSY and HMBC correlations. The carbon resonances of C-30 (*δ*_C_ 59.8, CH) and C-31 (*δ*_C_ 59.7, Cq) were attributed to epoxy carbons, while a hydroxy group linked to C-33 was confirmed by the HMBC correlations from H-33 (*δ*_H_ 4.26, dd, *J* = 2.0, 10.0 Hz) to C-21 and C-35 and the COSY relationship between OH (*δ*_H_ 4.63, br)/H-33. The NOE correlations between H_3_-39 (*δ*_H_ 1.52)/H_2_-25 (*δ*_H_ 2.00, 2.22) and H-22 (*δ*_H_ 4.70)/H_2_-24 (*δ*_H_ 1.92, 2.07) were in accordance with 22*E* and 26*E* geometries (Figure 2). The NOE relationships of H-33/H-30 (*δ*_H_ 2.30, m), H-33/H-22, and H-22/H-2 led to the assignment of β-faces of H-30 and H-33 and the *trans*-configuration of the epoxide group.

Lobophytone T (**6**) has a molecular formula of C_22_H_34_O_5_ as determined by HR-ESIMS (*m*/*z* 401.2292 [M + Na]^+^), implying six degrees of unsaturation. The ^1^H MMR displayed five methyl singlets including two olefinic methyls at *δ*_H_ 1.65 (3H, s) and 1.80 (3H, s) and an acetyl methyl group at *δ*_H_ 1.87 (3H, s), in addition to four olefinic protons for an AB spin system at *δ*_H_ 6.02 (d, *J* = 11.3 Hz) and 6.03 (d, *J* = 11.3 Hz) and for the *exo*-methylene at *δ*_H_ 4.86 (brs) and 4.82 (brs). The COSY correlations established the segments from C-2 to C-3, C-5 to C-7, C-9 to C-11, and C-13 to C-14, while ^13^C NMR and HMQC data indicated the methines of C-7 (*δ*_C_ 85.0), C-11 (*δ*_C_ 70.5) and C-14 (*δ*_C_ 69.7) bearing oxygen atom. The HMBC relationships connected each segment to form a backbone that featured a cembrane with 14-membered ring. An isopropene was deduced to position at C-1 based on the HMBC cross-peaks from H_2_-16 (*δ*_H_ 4.86, 4.82) and H_3_-17 (*δ*_H_ 1.80) to C-1 (*δ*_C_ 143.5), while additional HMBC correlations indicated two vinyl groups to be conjugated at C-1/C-2 and C-3/C-4. The HMBC correlations from H_3_-19 (*δ*_H_ 1.01) to C-7, C-8 (*δ*_C_ 69.2, Cq), and C-9 (*δ*_C_ 32.2), and from H_3_-20 (*δ*_H_ 1.05) to C-11, C-12 (*δ*_C_ 73.7, Cq), and C-13 (*δ*_C_ 44.3), in addition to the HMBC relationship from H-7 (*δ*_H_ 3.26) to C-11 established an ether bridge which linked C-7 and C-11. The acetoxy group was deduced to position at C-14 on the basis of the HMBC correlations from H-14 (*δ*_H_ 6.01) to the acetyl carbonyl carbon and to C-1, C-2, and also C-15 (*δ*_C_ 144.2, Cq). Thus, C-8 and C-12 were hydroxylated, and the gross structure of **6** was established as shown in Figure 1. The relative configurations of the chiral centers in **6** were determined through *J* values and NOESY analysis. The coupling constants of H-11 (*δ*_H_ 3.71, dd, *J* = 2.7, 11.7 Hz) suggested its axial orientation in the chair-conformation of the perhydropyrano ring, while the NOE relationship between H-11/H-14 indicated the same face (β-orientation) of both protons. The NOE interactions of OH-12 (*δ*_H_ 4.03, s) with H-11 and H-14 assigned to OH-12β, whereas the NOESY cross-peaks between H-11/H-9a (*δ*_H_ 1.50, m), H_3_-19/H-9a, and H-7/OH-8 (*δ*_H_ 3.95, s) suggested α-orientation for H-7 and OH-8.

The “monomeric” cembrane lobophytone T (**6**) is likely a precursor (diene) to form the right part of biscembranes when it reacted with methyl tortuosoate [6]. Thus, it provided new evidence to support the plausible biogenetic pathway to generate biscembranes. It is noted that all of the isolated biscembranoids (**1**–**5**) maintained the same partial structure of ring A, which was considered to be derived from methyl tortuosoate as a cembranoid-dienophile. The difference was found in ring C where epoxide rearrangement, oxidation, acetylation, and halogen substitution occurred.

In primary bioassays, compound **3** showed significant inhibition against lipopolysaccharide (LPS)-induced nitric oxide (NO) production in mouse peritoneal macrophages with IC_50_ = 2.8 μM, whereas the other compounds showed weak activity (IC_50_ > 10 μM). In addition, all compounds were weakly cytotoxic toward the mouse peritoneal macrophages (IC_50_ > 10 μM). The antibiotic assay indicated that compounds **3** and **6** exhibited strong inhibition against *Staphylococcus aureus*, *S. pneumoniae*, and *Saccharomyces cerevisiae* with the inhibitory rates around 90% at 20 μg/mL, but all compounds possessed weakly inhibitory activity against *Pseudomonas aerugonisa*, *Escherichia coli*, *Candida albicans*, and *Aspergillus fumigatus*.

## 3. Experimental Section

### 3.1. General Procedures

Optical rotations were measured on a Perkin-Elmer 243B polarimeter. IR spectra were determined on a Thermo Nicolet Nexus 470 FTIR spectrometer. ^1^H and ^13^C NMR and 2D NMR spectra were recorded on an Avance-500 FT 500 MHz NMR spectrometer using TMS as an internal standard. *δ* values are expressed in parts per million (ppm), and *J* values are reported in Hertz (Hz). HR-ESIMS data were obtained from Bruker APEX IV instrument. Low pressure column chromatography was carried out with silica gel (160–200 and 200–300 mesh), and GF_254_ silica gel for TLC was provided by Qingdao Marine Chemistry Co. Ltd. HPLC chromatography was performed on an Alltech instrument (426-HPLC pump, Alltech UV–vis-200 detector) equipped with Kromasil semipreparative column (10 μm, ODS, 10 mm × 250 mm) and YMC-Pack C_8_ (5 μm, 10 mm × 250 mm) column.

### 3.2. Animal Material

Soft coral *Lobophytum pauciflorum* was collected from the inner coral reef at a depth of 10 m in Sanya Bay, Hainan Island of China, in 2008. Fresh samples were frozen immediately. The specimen was identified by Leen van Ofwegen (National Museum of National History Naturalis). The coral specimen (HSF-6) was deposited at State Key Laboratory of Natural and Biomimetic Drugs, Peking University, China.

### 3.3. Extraction and Isolation

The frozen soft coral *L. pauciflorum* (2.3 kg) was homogenized and then extracted with EtOH (twice, 5 L/each time). The concentrated extract was desalted by dissolving in MeOH to yield a MeOH soluble fraction which was concentrated to an oily residue (92.7 g). This residue was partitioned between H_2_O and EtOAc, and then the water phase was extracted with *n*-BuOH. The EtOAc fraction (12.1 g) was subjected to silica gel (200–300 mesh) column chromatography, eluting with a gradient (petroleum ether–acetone, 20:1, 10:1, 3:1, 1:1) to obtain seven fractions (F1–F7). Fractions F1–F4 mainly contained lipids and steroids, as detected by ^1^H NMR, while F5 and F6 showed the ^1^H NMR spectral features of terpenoids. Thus, F5 and F6 were combined (1.69 g) and subsequently subjected to Sephadex LH-20 column eluting with CH_2_Cl_2_-MeOH (1:2) to afford 6 portions (P1–P6). P2 (180 mg) showing blue-green spots after spraying with anisaldehyde reagent was further separated on reversed-phase semi-preparative HPLC with CH_3_CN-H_2_O (61%) as a mobile phase to obtain **1** (12.5 mg). P3 (49.2 mg) was separated on ODS HPLC with MeOH–H_2_O (82%) as a mobile phase to afford **4** (7.6 mg), while **2** (8.1 mg) was obtained from P4 (40.5 mg) by the same separation process as that for P3. Followed by the same process as for P3 and P4, **5** (7.1 mg), **3** (17.7 mg), **3** (14.5 mg), and **6** (5.4 mg) were separated and purified from P5 (112.7 mg) by reversed-phase semi-preparative HPLC (ODS) with CH_3_CN–H_2_O (87%) as a mobile phase.

#### 3.3.1. Lobophytone O (**1**)

Amorphous powder; [α]_D_^25^+ 140.4 (*c* 1.25, CHCl_3_); IR (KBr) *ν*_max_ 3413, 2960, 2931, 1740, 1705, 1459, 1436, 1386, 1370, 1206, 1086, 1049, 1026 cm^−1; 1^H and ^13^C NMR data, see Tables 1 and 2; HR-ESIMS *m/z* 723.4428 [M + Na]^+^ (calcd for C_41_H_64_NO_9_Na, 723.4442).

#### 3.3.2. Lobophytone P (**2**)

Amorphous powder; [α]_D_^25^ + 133.7 (*c* 0.33, CHCl_3_); IR (KBr) *ν*_max_ 3467, 2959, 2928, 1741, 1708, 1459, 1373, 1237, 1028 cm^−1; 1^H and ^13^C NMR data, see Tables 1 and 2; HR-ESIMS *m/z* 765.4570 [M + Na]^+^ (calcd for C_43_H_66_O_10_Na, 765.4548).

#### 3.3.3. Lobophytone Q (**3**)

Amorphous powder; [α]_D_^25^ + 121.2 (*c* 1.30, CHCl_3_); IR (KBr) *ν*_max_ 3440, 2958, 2930, 1741, 1706, 1459, 1374, 1206, 1027 cm^−1; 1^H and ^13^C NMR data, see Tables 1 and 2; HR-ESIMS *m/z* 741.4096 [M + Na]^+^ (calcd for C_41_H_63_O_8_NaCl, 741.4104).

#### 3.3.4. Lobophytone R (**4**)

Amorphous powder; [α]_D_^25^ + 151.1 (*c* 0.51, CHCl_3_); IR (KBr) *ν*_max_ 3423, 2958, 2928, 1709, 1460, 1376, 1203, 1050 cm^−1; 1^H and ^13^C NMR data, see Tables 1 and 2; HR-ESIMS *m/z* 685.4670 [M + H]^+^ (calcd for C_41_H_65_O_8_, 685.4674).

#### 3.3.5. Lobophytone S (**5**)

Amorphous powder; [α]_D_^25^ + 101.1 (*c* 0.60, CHCl_3_); IR (KBr) *ν*_max_ 3399, 2958, 2927, 1742, 1706, 1459, 1435, 1387, 1204, 1074 cm^−1; 1^H and ^13^C NMR data, see Tables 1 and 2; HR-ESIMS *m/z* 689.4384 [M + Na]^+^ (calcd for C_41_H_62_O_7_Na, 689.4388).

#### 3.3.6. Lobophytone T (**6**)

Colorless oil; [α]_D_^25^ + 36.5 (*c* 0.27, CHCl_3_); IR (KBr) *ν*_max_ 3423, 2969, 2935, 1734, 1453, 1375, 1246, 1071, 1026 cm^−1; 1^H NMR *δ* (ppm, DMSO-*d*_6_): 6.02 (1H, d, *J* = 11.3 Hz, H-2), 6.03 (1H, d, *J* = 11.3 Hz, H-3), 2.47 (1H, m, H-5a), 1.96 (1H, m, H-5b), 1.44 (1H, m, H-6a), 2.00 (1H, m, H-6b), 3.26 (1H, brd, *J* = 8.1 Hz, H-7), 1.42 (1H, m, H-9a), 1.50 (1H, m, H-9b), 1.43 (1H, m, H-10a), 1.77 (1H, m, H-10b), 3.71 (1H, dd, *J* = 2.7, 11.7 Hz, H-11), 1.85 (1H, m, H-13a), 1.97 (1H, m, H-13b), 6.01 (1H, brd, *J* = 10.0 Hz, H-14), 4.82 (1H, br, H-16a), 4.86 (1H, br, H-16b), 1.80 (3H, s, H-17), 1.65 (3H, s, H-18), 1.01 (3H, s, H-19), 1.05 (3H, s, H-20), 1.87 (3H, s, Ac); ^13^C NMR *δ* (ppm, DMSO-*d*_6_): 143.5 (Cq, C-1), 120.5 (CH, C-2), 119.6 (CH, C-3), 137.8 (Cq, C-4), 39.4 (CH_2_, C-5), 21.6 (CH_2_, C-6), 85.0 (CH, C-7), 69.2 (Cq, C-8), 32.2 (CH_2_, C-9), 19.9 (CH_2_, C-10), 70.5 (CH, C-11), 73.7 (Cq, C-12), 44.3 (CH_2_, C-13), 69.7 (CH, C-14), 144.2 (Cq, C-15), 114 (CH_2_, C-16), 23.0 (CH_3_, C-17), 18.1 (CH_3_, C-18), 27.8 (CH_3_, C-19), 25.0 (CH_3_, C-20), 169.9 (Cq, Ac), 21.5 (CH_3_, Ac); HR-ESIMS *m/z* 401.2292 [M + Na]^+^ (calcd for C_22_H_34_O_5_Na, 401.2298).

### 3.4. Assay for Inhibition of Nitric Oxide (NO) Production

Dexamethasone (DEX, positive control, 20 mM in DMSO) and each compound (20 mM in DMSO) were diluted to 1–20 μM range at r.t. before experiment. The final percentage of DMSO in the reaction mixture was less than 0.5% (v/v). LPS (1 μg/mL), 4% sodium thioglycolate, RPMI1640, FBS, PBS, MTT and Griess reagents were purchased from Sigma (St. Louis, MO, U.S.). Mouse peritoneal macrophages (PEMΦ) were obtained from C57BL6J male mice, and then plated onto 48 well plates and cultured for 2 hrs in DMEM containing 5% FBS at 5% CO_2_ in 37 °C. Mouse PEMΦ were incubated with compounds for 1 h at 37 °C before stimulation with 1 μg/mL of lipopolysaccharide (LPS) for 24 h. Cells (5 × 10^5^ cells) were preincubated at 37 °C for 24 h in serum-free medium and NO production was monitored by measuring nitrite levels in culture media using Griess reagent. Absorbance was measured at 548 nm in incubated media with Griess reagent for 10 min. Viable adherent cells were stained with MTT (2 μg/mL) for 4 h. The medium was then removed and the produced formazan crystals were dissolved in DMSO (200 μL). Absorbance was measured at 540 nm.

One-way analysis of variance was applied for all statistical analyses by independent experiments, and data were represented as means ± standard error of the measurement. Individual values were compared by t-test and a P-value < 0.01 to evaluate the significant.

### 3.5. Antimicrobial and Antifungal Assays

Antimicrobial and antifungal bioassays were conducted in triplicate following the method as recommended by the National Center for Clinical Laboratory Standards (NCCLS) [10]. The bacterial strains *Staphylococcus aureus*, *S. pneumoniae*, *Escherichia coli*, and *Pseudomonas aeruginosa* were grown on Mueller-Hinton agar. The yeasts, *Candida albicans* and *Saccharomyces cerevisiae*, were grown on Sabouraud dextrose agar, and the fungus, *Aspergillus fumigatus*, was grown on potato dextrose agar. Targeted microbes (3–4 colonies) were prepared from broth culture (bacteria: 37 °C for 24 h; fungus: 28 °C for 48 h), and the final spore suspensions of bacteria (in MHB medium), yeasts (in SDB medium), and fungus (in PDB medium) were 10^6^ and 10^5^ cells/mL and 10^4^ mycelial fragments/mL, respectively. Testing compounds (10 mg/mL as stock solution in DMSO and serial dilutions) were transferred to a 96-well clear plate in triplicate, and the suspension of the test microorganisms were added to each well (200 μL) (antimicrobial peptide AMP, streptomycin, and fluconazole were used as positive controls). After incubation, the absorbance at 595 nm was measured with a microplate reader (TECAN), and the inhibition rate was calculated and plotted *versus* test concentrations.

## Supplementary Material



## Figures and Tables

**Table 1:** ^13^C NMR data of lobophytones O–S (1–5) (*δ*39.5, DMSO-*d*_6_).

Position	1	2	3	4	5
1	49.4 (Cq)	49.4 (Cq)	49.3 (Cq)	48.5 (Cq)	49.9 (Cq)
2	43.9 (CH)	43.8 (CH)	43.9 (CH)	45.9 (CH)	44.0 (CH)
3	213.8 (Cq)	213.8 (Cq)	213.8 (Cq)	212.1 (Cq)	213.9 (Cq)
4	54.1 (CH_2_)	54.0 (CH_2_)	54.1 (CH_2_)	50.4 (CH_2_)	53.9 (CH_2_)
5	27.3 (CH)	27.3 (CH)	27.3 (CH)	26.9 (CH)	27.3 (CH)
6	38.0 (CH_2_)	37.9 (CH_2_)	38.0 (CH_2_)	37.0 (CH_2_)	38.0 (CH_2_)
7	26.0 (CH_2_)	26.0 (CH_2_)	26.0 (CH_2_)	24.4 (CH_2_)	26.0 (CH_2_)
8	34.1 (CH_2_)	34.0 (CH_2_)	34.1 (CH_2_)	32.7 (CH_2_)	34.1 (CH_2_)
9	47.6 (CH)	47.5 (CH)	47.7 (CH)	46.4 (CH)	47.8 (CH)
10	213.3 (Cq)	213.3 (Cq)	213.3 (Cq)	213.6 (Cq)	213.1 (Cq)
11	30.9 (CH_2_)	31.0 (CH_2_)	30.9 (CH_2_)	34.9 (CH_2_)	30.9 (CH_2_)
12	51.8 (CH)	51.8 (CH)	51.7 (CH)	51.7 (CH)	51.3 (CH)
13	210.0 (Cq)	209.9 (Cq)	209.9 (Cq)	211.5 (Cq)	210.3 (Cq)
14	45.9 (CH_2_)	45.8 (CH_2_)	45.9 (CH_2_)	46.9 (CH_2_)	46.2 (CH_2_)
15	28.7 (CH)	28.7 (CH)	28.7 (CH)	28.8 (CH)	28.9 (CH)
16	21.5 (CH_3_)	21.5 (CH_3_)	21.5 (CH_3_)	21.2 (CH_3_)	21.5 (CH_3_)
17	17.6 (CH_3_)	17.6 (CH_3_)	17.6 (CH_3_)	18.3 (CH_3_)	17.7 (CH_3_)
18	17.6 (CH_3_)	17.6 (CH_3_)	17.6 (CH_3_)	17.1 (CH_3_)	17.7 (CH_3_)
19	22.9 (CH_3_)	22.9 (CH_3_)	22.9 (CH_3_)	22.1 (CH_3_)	22.8 (CH_3_)
20	174.8 (Cq)	174.7 (Cq)	174.7 (Cq)	173.8 (Cq)	174.6 (Cq)
21	43.7 (CH)	43.5 (CH)	43.7 (CH)	50.2 (CH)	43.2 (CH)
22	128.6 (CH)	129.4 (CH)	130.0 (CH)	124.6 (CH)	124.6 (CH)
23	137.8 (Cq)	136.9 (Cq)	136.2 (Cq)	135.9 (Cq)	133.7 (Cq)
24	33.5 (CH_2_)	32.9 (CH_2_)	34.0 (CH_2_)	36.7 (CH_2_)	37.3 (CH_2_)
25	33.1 (CH_2_)	31.1 (CH_2_)	34.8 (CH_2_)	24.4 (CH_2_)	23.8 (CH_2_)
26	70.4 (CH)	73.5 (CH)	65.1 (CH)	77.8 (CH)	125.1 (CH)
27	85.1 (Cq)	83.2 (Cq)	84.8 (Cq)	74.7 (Cq)	132.8 (Cq)
28	35.9 (CH_2_)	36.1 (CH_2_)	36.8 (CH_2_)	39.7 (CH_2_)	35.6 (CH_2_)
29	28.1 (CH_2_)	28.0 (CH_2_)	28.0 (CH_2_)	27.6 (CH_2_)	25.1 (CH_2_)
30	89.2 (CH)	89.6 (CH)	90.1 (CH)	80.0 (CH)	59.8 (CH)
31	73.6 (Cq)	73.3 (Cq)	73.2 (Cq)	80.5 (Cq)	59.7 (Cq)
32	40.8 (CH_2_)	41.5 (CH_2_)	40.7 (CH_2_)	41.5 (CH_2_)	39.6 (CH_2_)
33	64.0 (CH)	64.1 (CH)	63.9 (CH)	24.4 (CH_2_)	62.6 (CH)
34	135.6 (Cq)	135.2 (Cq)	135.2 (Cq)	132.8 (Cq)	133.1 (Cq)
35	123.2 (Cq)	123.2 (Cq)	123.6 (Cq)	123.3 (Cq)	127.6 (Cq)
36	32.5 (CH_2_)	32.6 (CH_2_)	32.5 (CH_2_)	32.1 (CH_2_)	32.6 (CH_2_)
37	18.4 (CH_3_)	18.4 (CH_3_)	18.3 (CH_3_)	20.1 (CH_3_)	18.7 (CH_3_)
38	20.6 (CH_3_)	20.3 (CH_3_)	20.2 (CH_3_)	17.9 (CH_3_)	17.6 (CH_3_)
39	20.3 (CH_3_)	20.8 (CH_3_)	20.3 (CH_3_)	25.4 (CH_3_)	17.3 (CH_3_)
40	23.5 (CH_3_)	23.4 (CH_3_)	23.5 (CH_3_)	15.5 (CH_3_)	20.3 (CH_3_)
OMe	51.2 (CH_3_)	51.1 (CH_3_)	51.2 (CH_3_)	51.0 (CH_3_)	51.2 (CH_3_)
Ac		171.3 (Cq)			
		21.4 (CH_3_)			

**Table 2:** ^1^H NMR data of lobophytones O–S (1–5) (*δ*2.50, DMSO-*d*_6_).

Position	1	2	3	4	5
2	3.47 t (8.3)	3.45 m	3.47 t (8.8)	3.49 m	3.59 t (8.1)
4	3.09 dd (10.3, 19.6)	3.09 dd (10.3, 19.5)	3.09 dd (10.3, 20.0)	2.61 dd (6.9, 17.8)	3.06 dd (10.3, 19.6)
	2.40 d (19.6)	2.40 d (19.5)	2.40 d (20.0)	2.32 m	2.44 d (19.6)
5	1.68 m	1.69 m	1.67 m	1.85 m	1.63 m
6	1.01 m	1.01 m	0.97 m	1.03 m	0.96 m
	1.07 m	1.06 m		0.87 m	1.00 m
7	1.10 m	1.12 m	1.08 m	0.95 m	0.94 m
	1.01 m	1.00 m	1.00 m		1.07 m
8	1.43 m	1.49 m	1.43 m	1.40 m	1.38 m
	1.36 m	1.35 m	1.35 m		1.40 m
9	2.33 m	2.33 m	2.32 m	2.38 m	2.30 m
11	2.80 dd (10.3, 16.6)	2.80 m	2.80 dd (10.5, 16.9)	2.73 dd (2.7, 16.9)	1.88 d (20.0)
	1.84 d (16.6)	1.84 m	1.83 d (16.9)	2.15 dd (2.4, 16.9)	2.82 dd (10.5, 20.0)
12	2.88 m	2.87 m	2.89 m	2.89 m	2.89 brd (10.5)
14	2.86 d (19.3)	2.88 d (19.3)	2.86 d (19.6)	2.96 m	2.94 d (19.6)
	2.72 d (19.3)	2.74 d (19.3)	2.72 d (19.6)	2.90 m	2.69 d (19.6)
15	2.33 m	2.34 m	2.32 m	2.07 m	2.22 m
16	0.95 d (6.7)	0.96 d (6.6)	0.95 d (6.9)	0.91 d (6.9)	0.92 d (6.6)
17	0.63 d (6.7)	0.63 d (6.6)	0.63 d (6.9)	0.71 d (6.9)	0.61 d (6.6)
18	1.05 d (6.9)	1.05 d (6.6)	1.05 d (7.0)	1.07 d (7.1)	1.04 d (6.9)
19	0.81 d (6.9)	0.81 d (6.6)	0.81 d (6.9)	0.81 d (6.9)	0.81 d (6.6)
21	3.38 d (10.5)	3.47 d (10.3)	3.38 d (10.3)	3.23 m	3.20 d (11.0)
22	4.78 d (10.5)	4.85 d (10.3)	4.87 d (10.3)	4.94 d (11.5)	4.70 d (11.0)
24	2.02 m	2.13 m	2.18 m	2.38 m	2.07 m
	1.91 m	1.51 m	1.96 m	1.87 m	1.92 m
25	1.65 m	1.80 m	2.08 m	1.82 m	2.22 m
	1.36 m	1.60 m	1.66 m	1.63 m	2.00 m
26	3.01 brd (10.5)	4.56 d (10.8)	3.55 d (11.7)	3.39 m	4.72 br
28	2.25 m	1.71 m	2.32 m	1.58 m	2.10 m
	1.38 m	1.45 m	1.62 m	1.42 m	1.91 m
29	1.67 m	1.71 m	1.75 m	1.85 m	1.76 m
	1.36 m	1.43 m	1.40 m	1.31 m	1.47 m
30	3.77 dd (5.3, 10.5)	3.80 dd (5.1, 10.5)	3.86 dd (5.4, 11.5)	2.91 m	2.30 m
32	1.65 m	1.71 m	1.62 m	2.07 m	1.96 dd (10.0, 15.4)
	1.09 m	1.13 m	1.09 m	1.04 m	1.70 dd (2.0, 15.4)
33	4.80 m	4.82 m	4.82 brt	2.10 m	4.26 dd (2.0, 10.0)
				1.83 m	
36	2.18 dd (8.3, 19.3)	2.21 dd (8.8, 19.3)	2.21 m	2.47 m	2.12 d (8.1)
	2.06 dd (8.8, 19.3)	2.03 m	2.05 m	1.98 dd (5.4, 18.3)	2.12 d (8.1)
37	1.66 s	1.67 s	1.67 s	1.54 s	1.63 s
38	1.65 s	1.64 s	1.67 s	1.69 s	1.53 s
39	0.99 s	1.09 s	1.19 s	0.98 s	1.52 s
40	0.97 s	0.99 s	0.99 s	0.92 s	1.06 s
OMe	3.42 s	3.39 s	3.42 s	3.39 s	3.40 s
Ac		2.06 s			
OH-26	4.53 br				
OH-27				4.18 s	
OH-30				4.49 br	
OH-31	3.98 s	4.14 s	4.21 s		
OH-33	4.40 br	4.45 br	4.46 br		4.63 br
