# Supplementary material for: Lobophytones O–T, New Biscembranoids and Cembranoid from Soft Coral Lobophytum pauciflorum
Source: Mar Drugs. 2010 Nov 10;8(11):2837–48. doi: 10.3390/md8112848 (PMC2996179; doi:10.3390/md8112848)
Supplement: Supplementary file 1 [file marinedrugs-08-02837-s001.pdf]

Lobophytones O–T, New Biscembranoids and Cembranoid from Soft Coral *Lobophytum pauciflorum*

Pengcheng Yan [1], Zhiwei Deng [2], Leen van Ofwegen [3], Peter Proksch [4], Wenhan Lin [1,*]

[1] State Key Laboratory of Natural and Biomimetic Drugs, Peking University, Beijing 100083, China; E-Mail: ypc@bjmu.edu.cn

[2] Analytical and testing Center, Beijing Normal University, Beijing, 100875, China; E-Mail: dengzw@bnu.edu.cn

[3] National Museum of Natural History Naturalis, 2300 RA Leiden, The Netherlands; E-Mail: ofwegen@naturalis.nnm.nl

[4] Institute of Pharmaceutical Biology and Biotechnology, Heinrich-Heine University, 40225 Duesseldorf, Germany; E-Mail: proksch@duesseldorf-uni.de

Supplemental Information

Spectra of compound 1

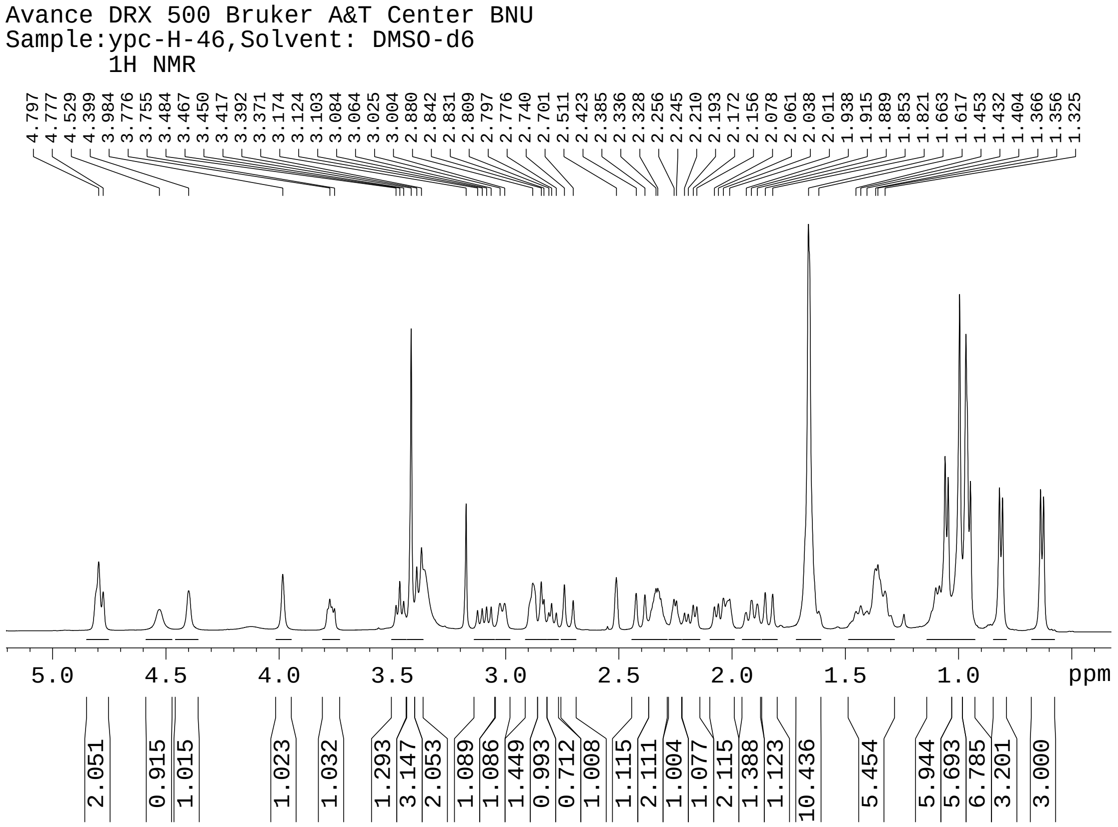

Figure S1 ^{1}H NMR spectrum of compound **1**

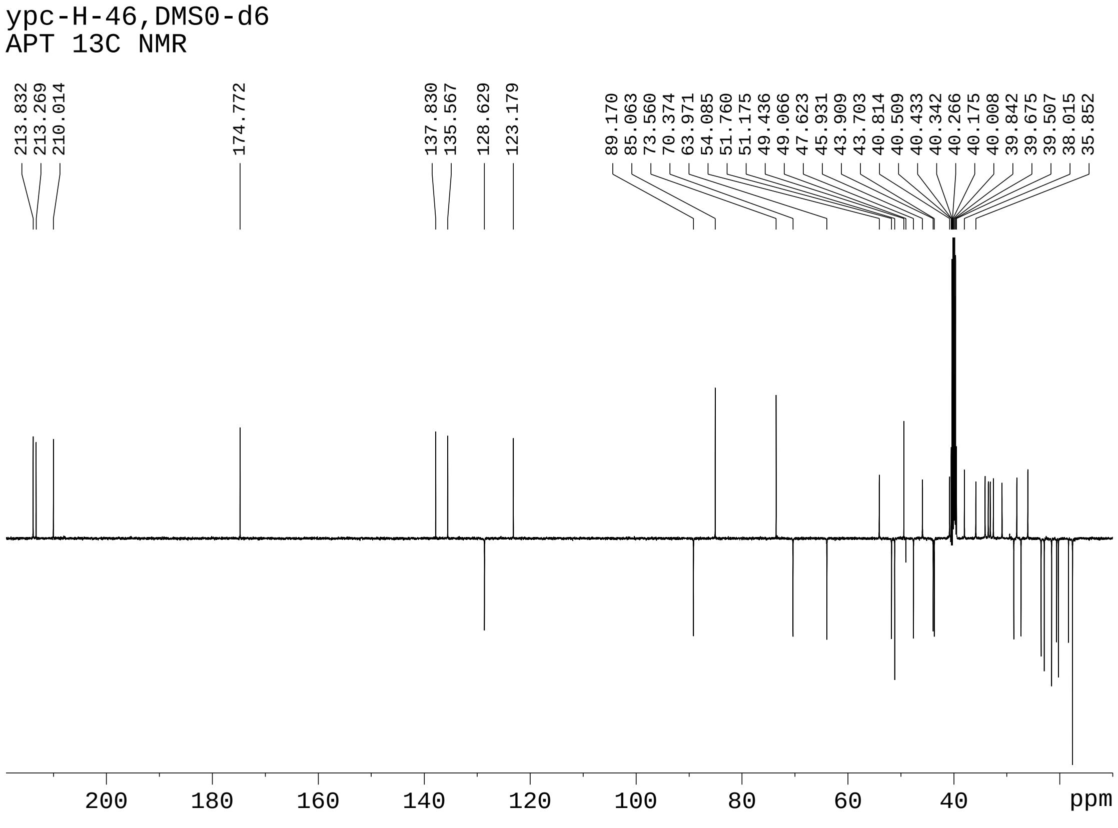

Figure S2 ^{13}C NMR spectrum of compound **1**

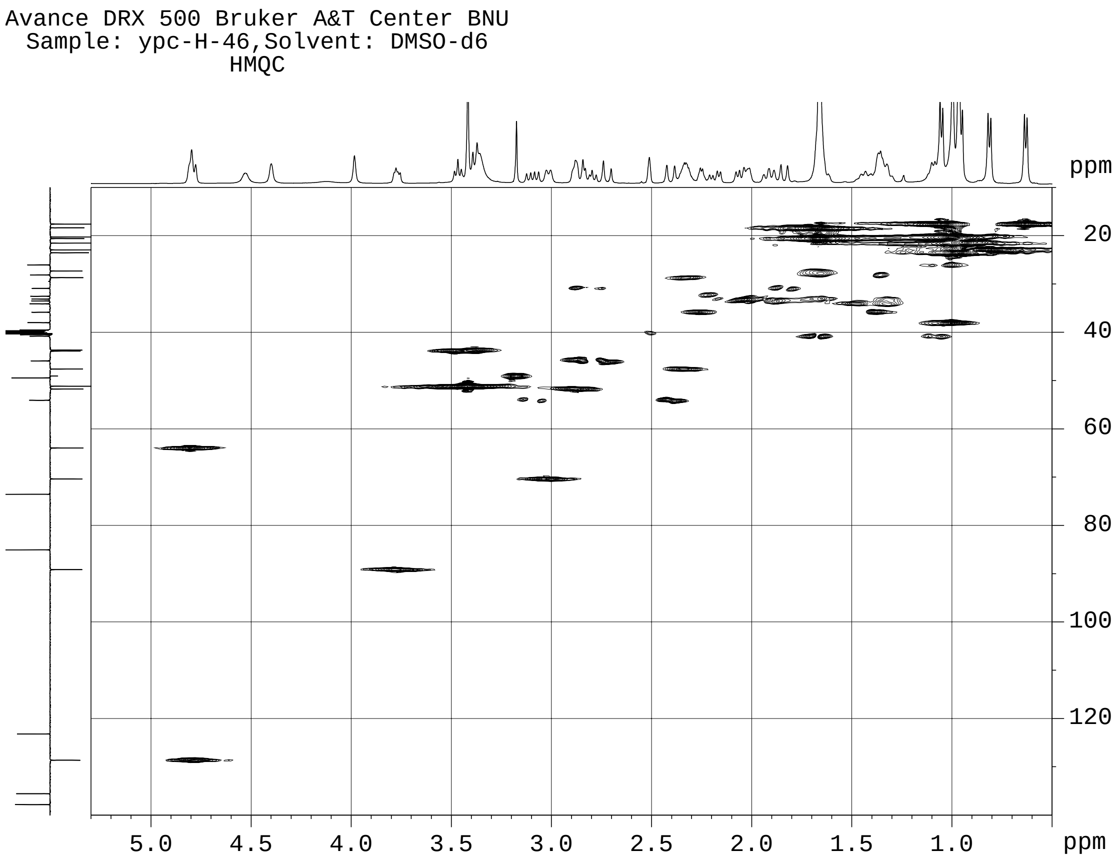

Figure S3 HMQC spectrum of compound **1**

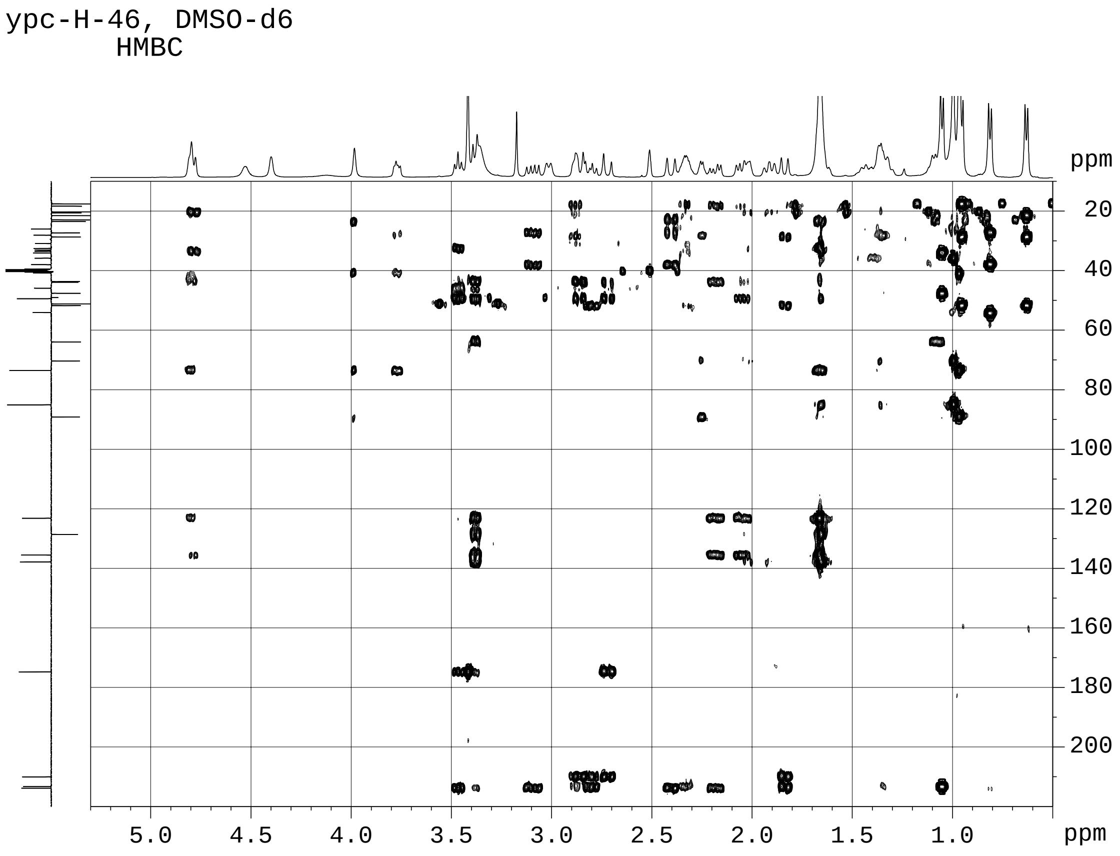

Figure S4 HMBC spectrum of compound **1**

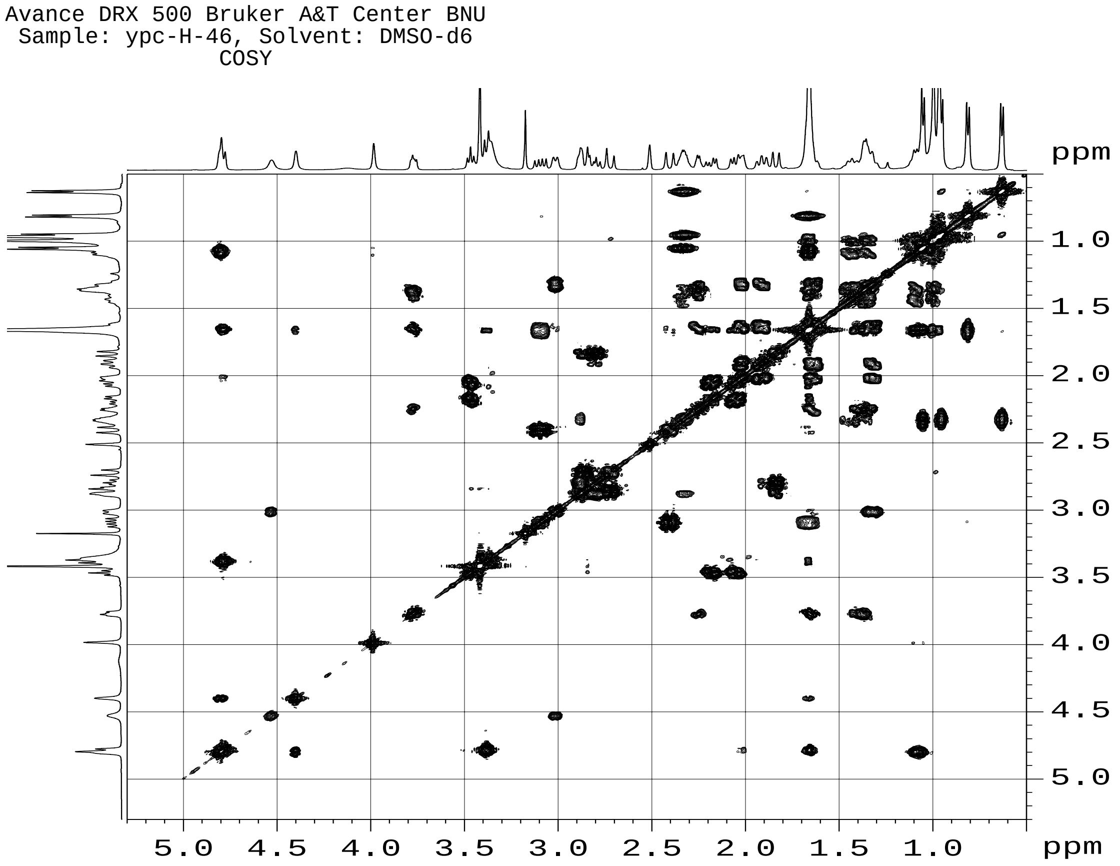

Figure S5 COSY spectrum of compound **1**

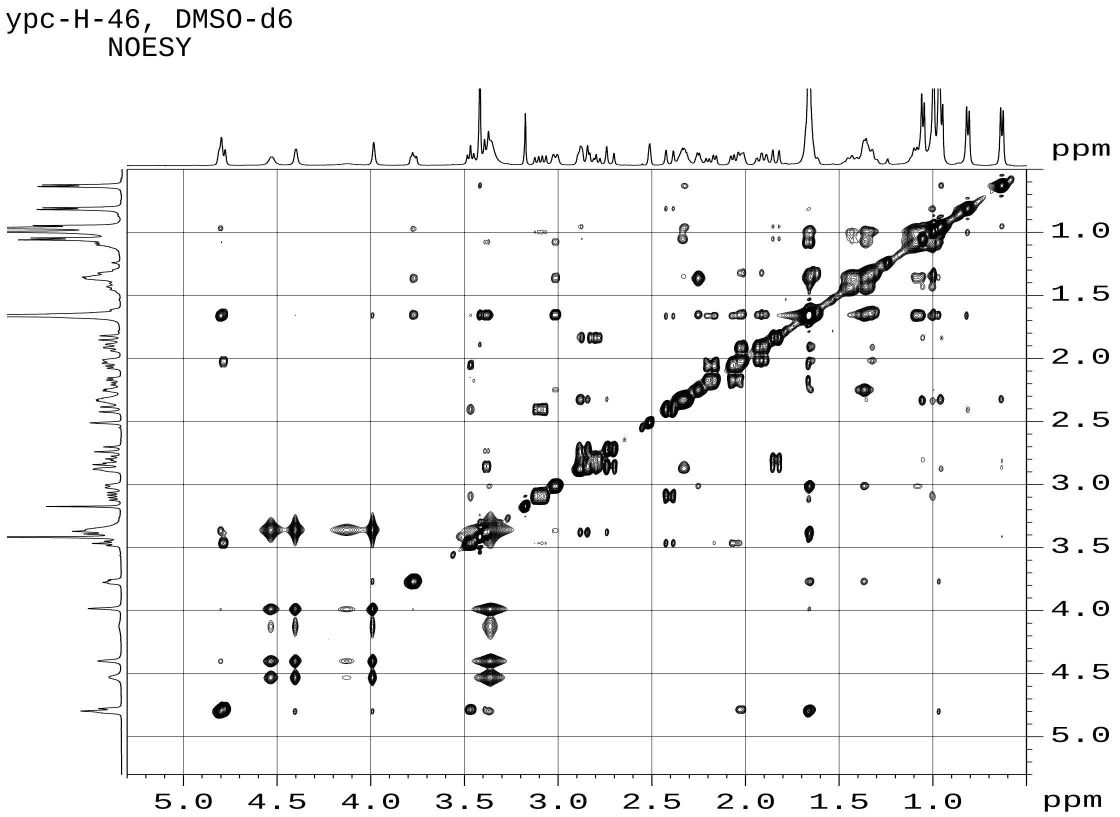

Figure S6 NOESY spectrum of compound **1**

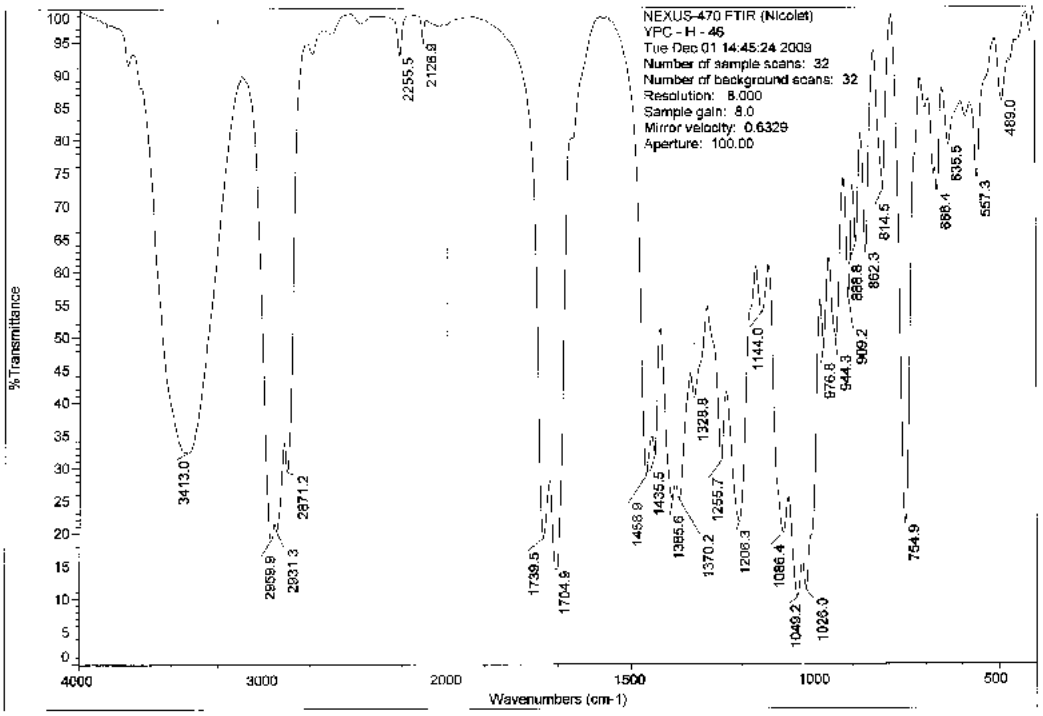

Figure S7 IR spectrum of compound **1**

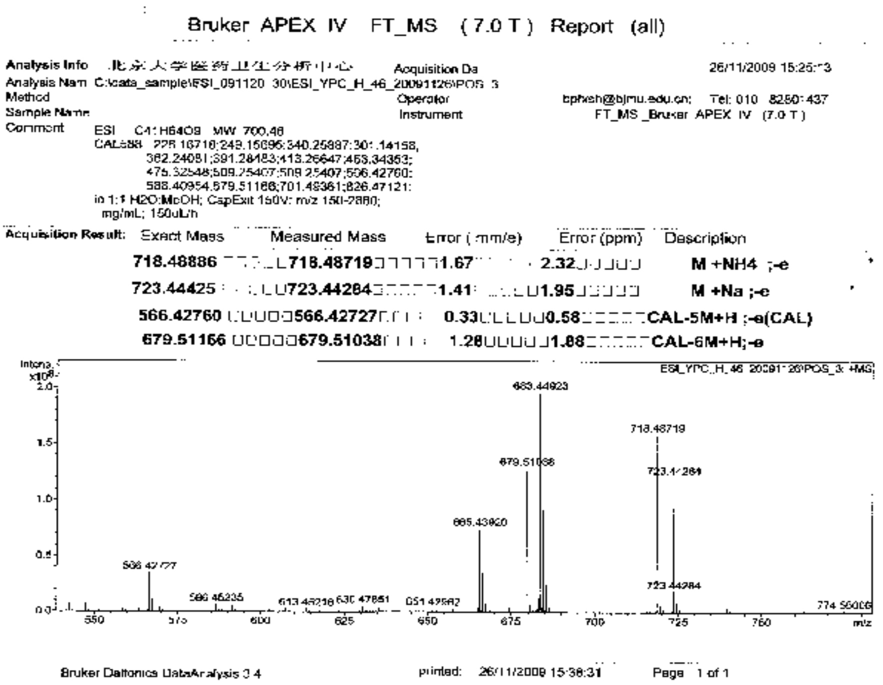

Figure S8 HRESIMS spectrum of compound **1**

Spectra of compound 2

Avance DRX 500 Bruker A&T Center BNU
Sample:ypc-H-20a, Solvent: DMSO-d6
1H NMR

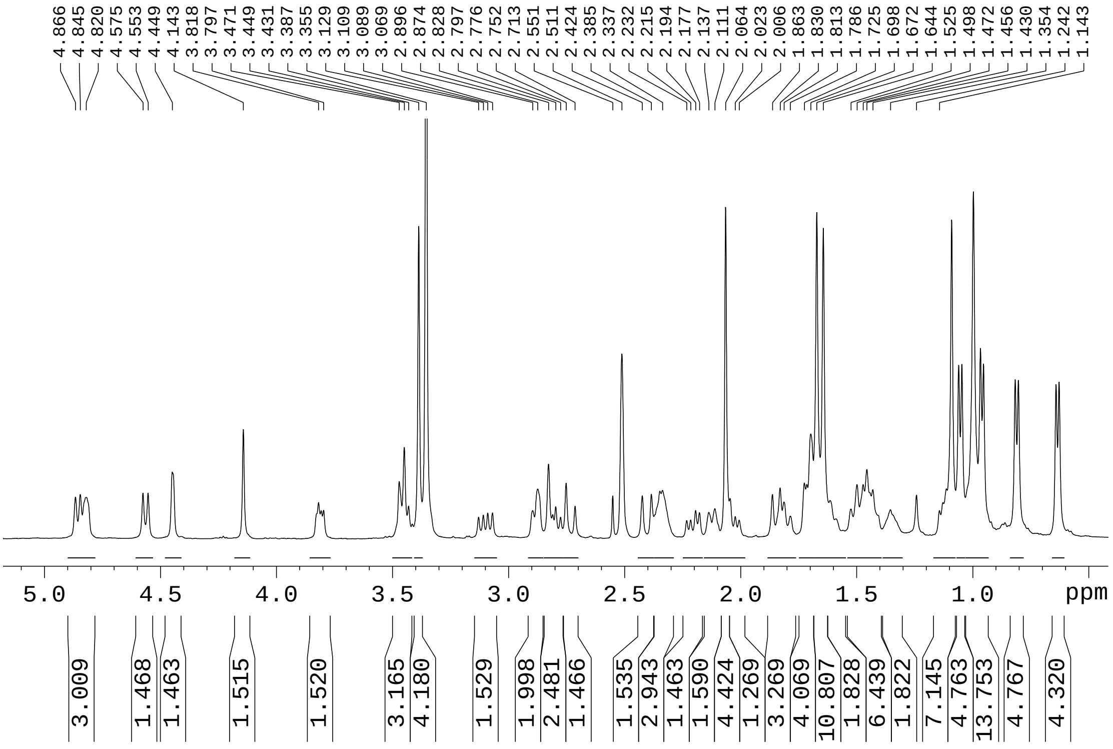

Figure S9 ^{1}H NMR spectrum of compound **2**

ypc-H-20a,DMS0-d6
APT 13C NMR

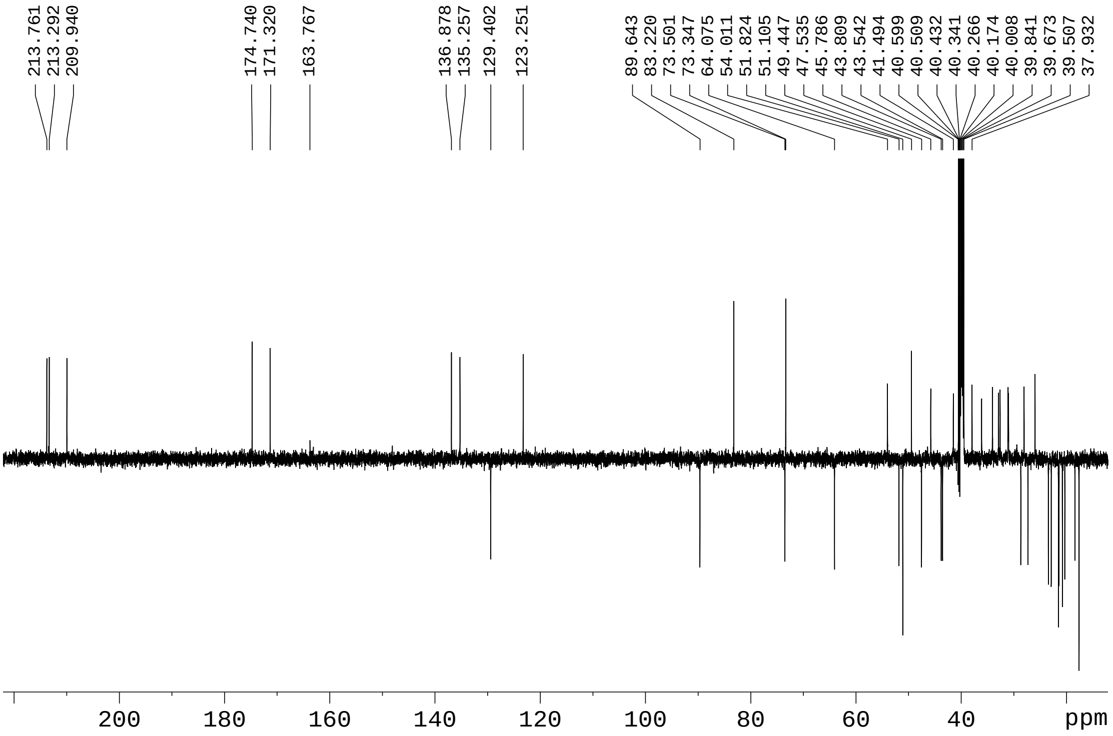

Figure S10 ^{13}C NMR spectrum of compound **2**

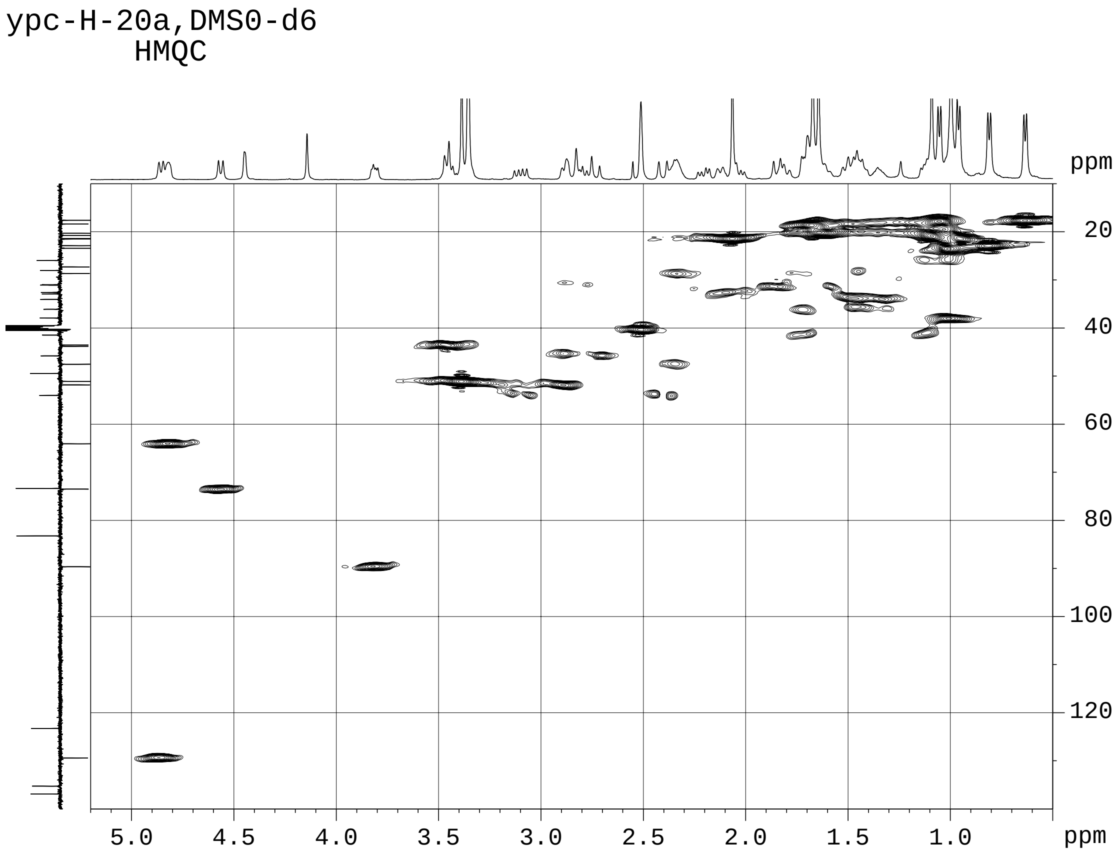

Figure S11 HMQC spectrum of compound **2**

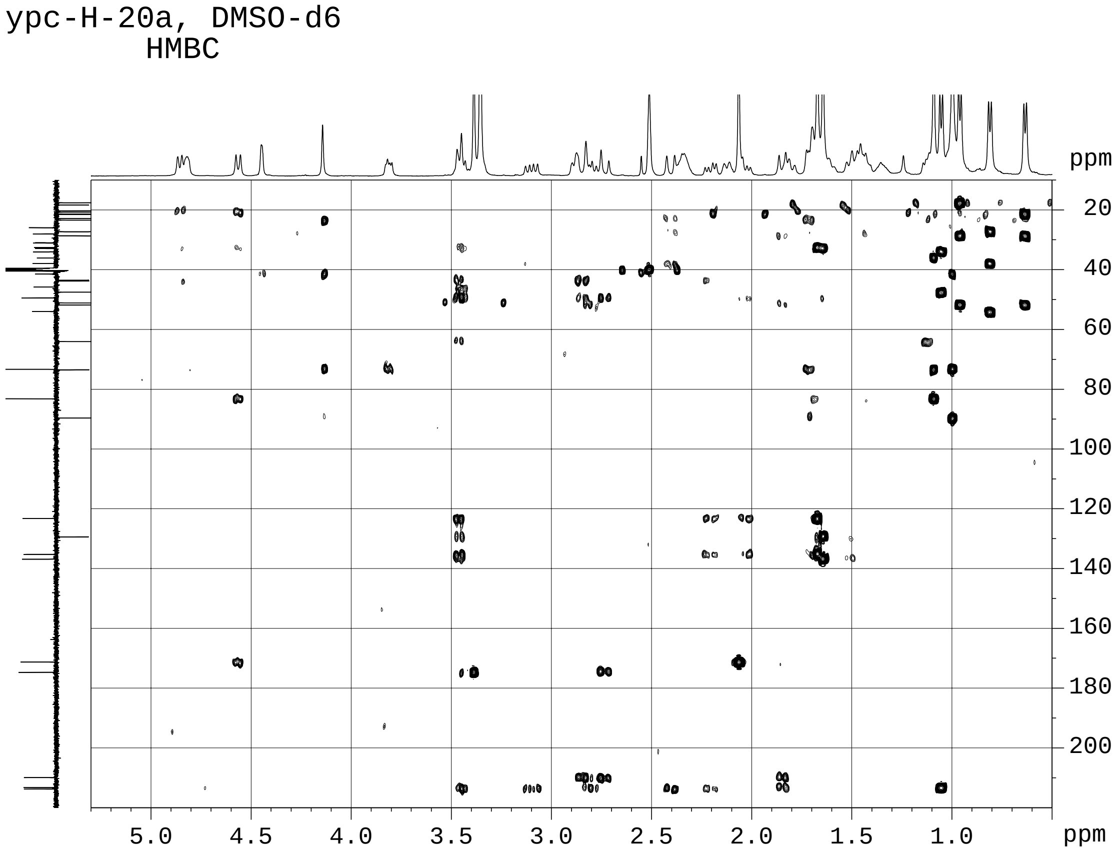

Figure S12 HMBC spectrum of compound **2**

Sample:ypc-H-20a, Solvent:DMSO-d6
COSY

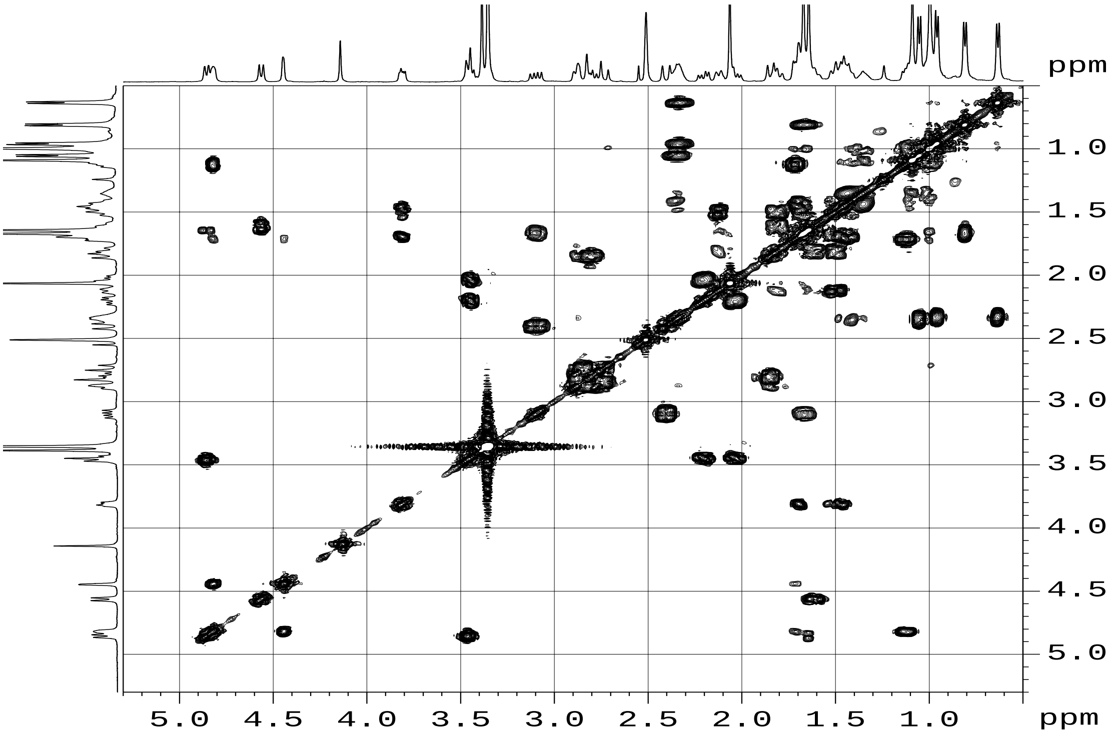

Figure S13 COSY spectrum of compound **2**

ypc-H-20a, DMSO-d6
NOESY

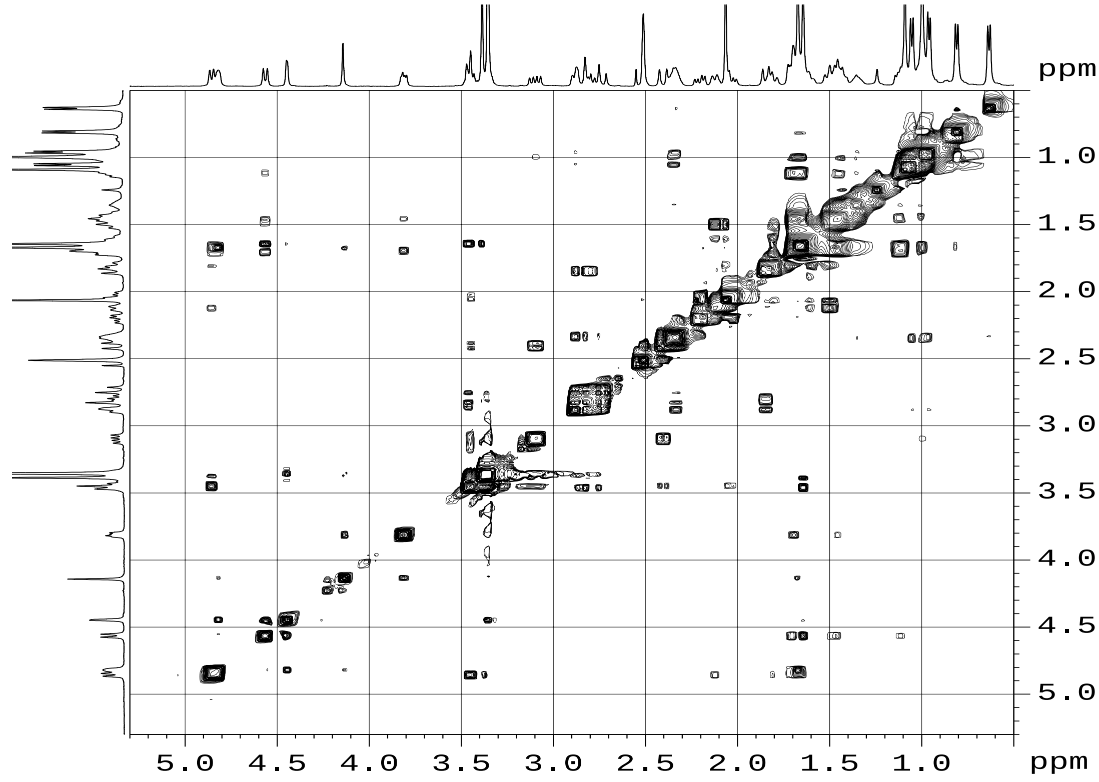

Figure S14 NOESY spectrum of compound **2**

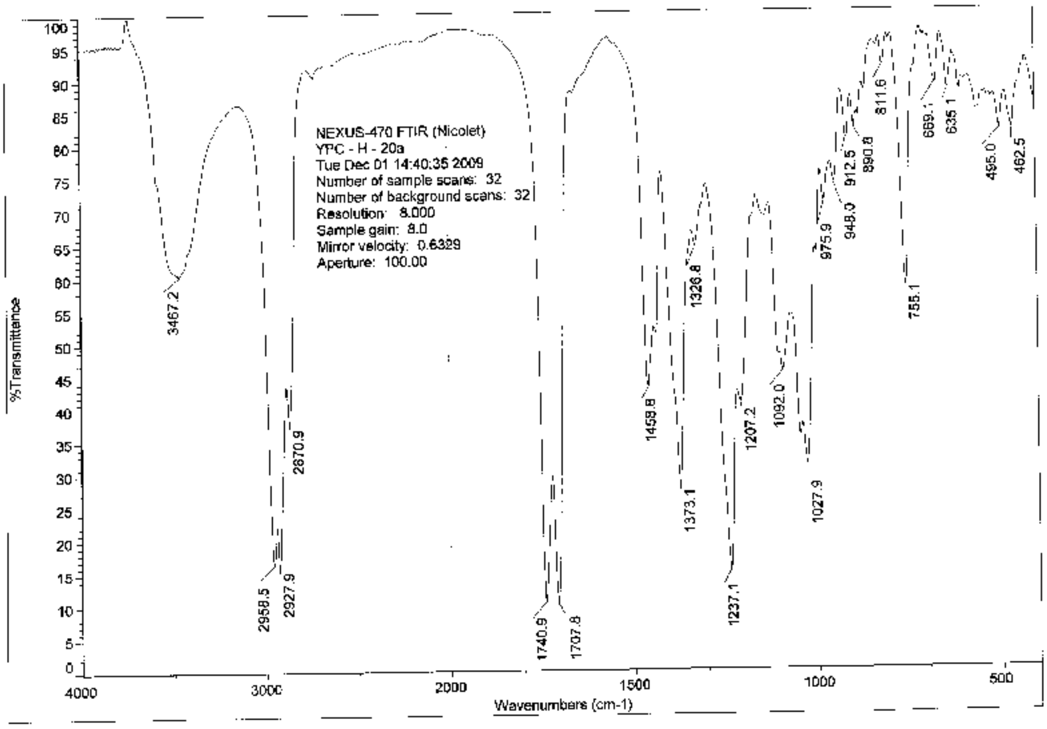

Figure S15 IR spectrum of compound **2**

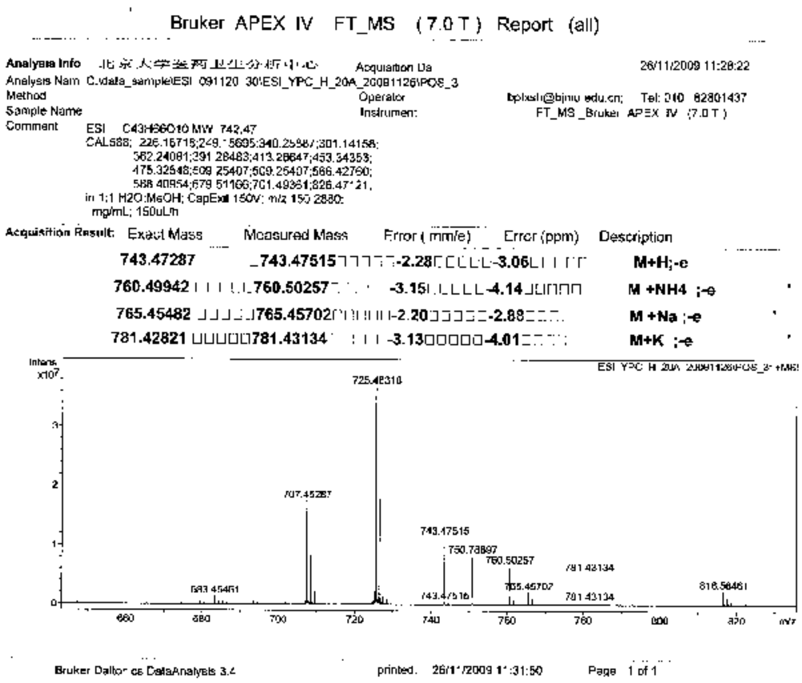

Figure S16 HRESIMS spectrum of compound **2**

Spectra of compound 3

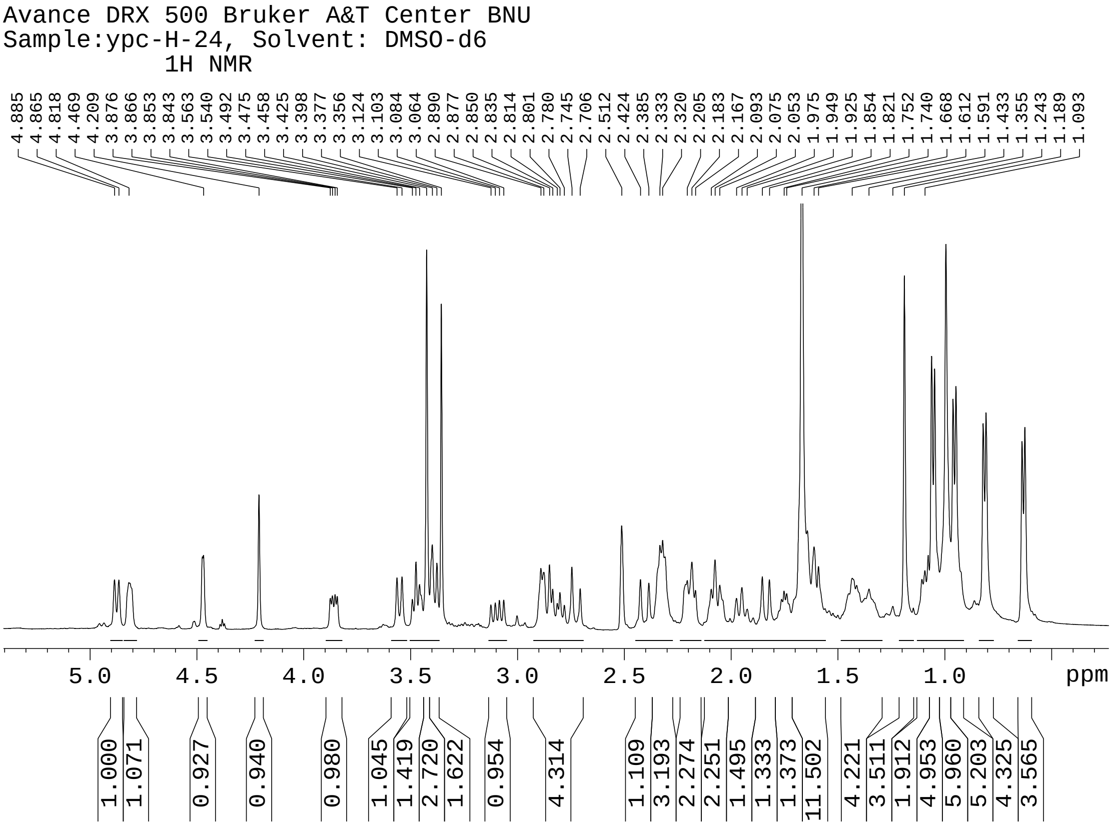

Figure S17 ^{1}H NMR spectrum of compound **3**

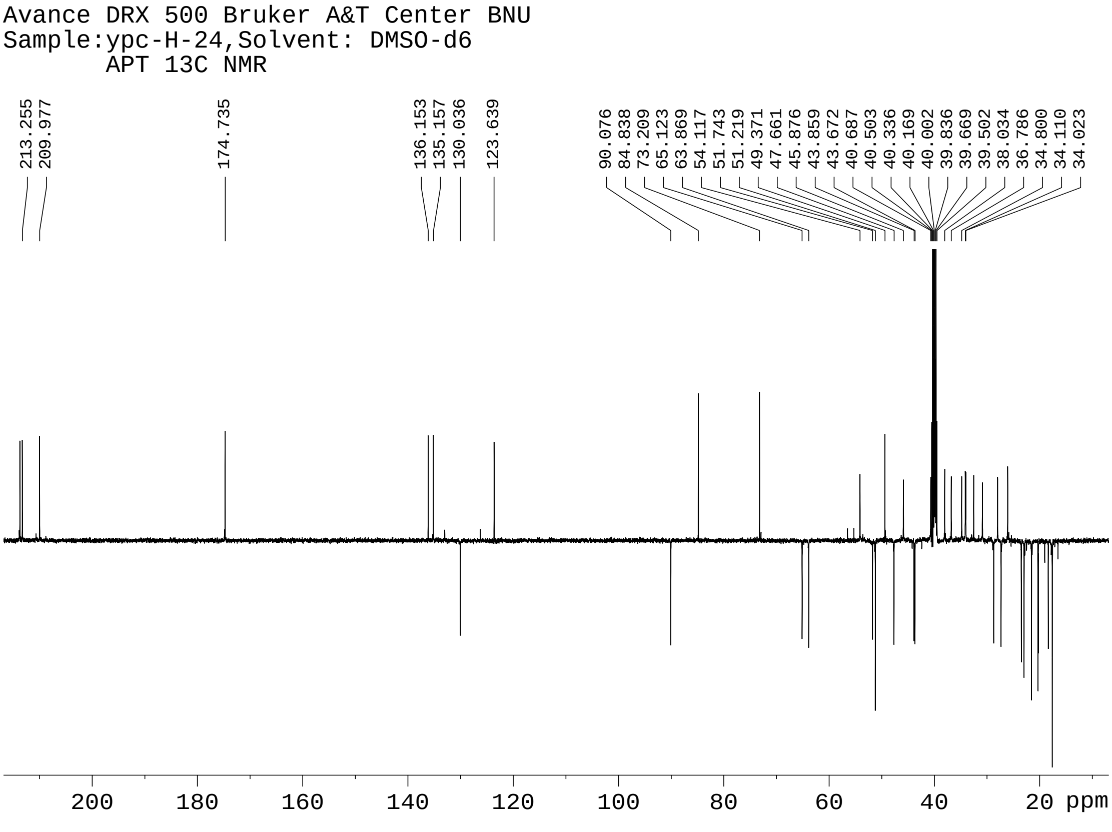

Figure S18 ^{13}C NMR spectrum of compound **3**

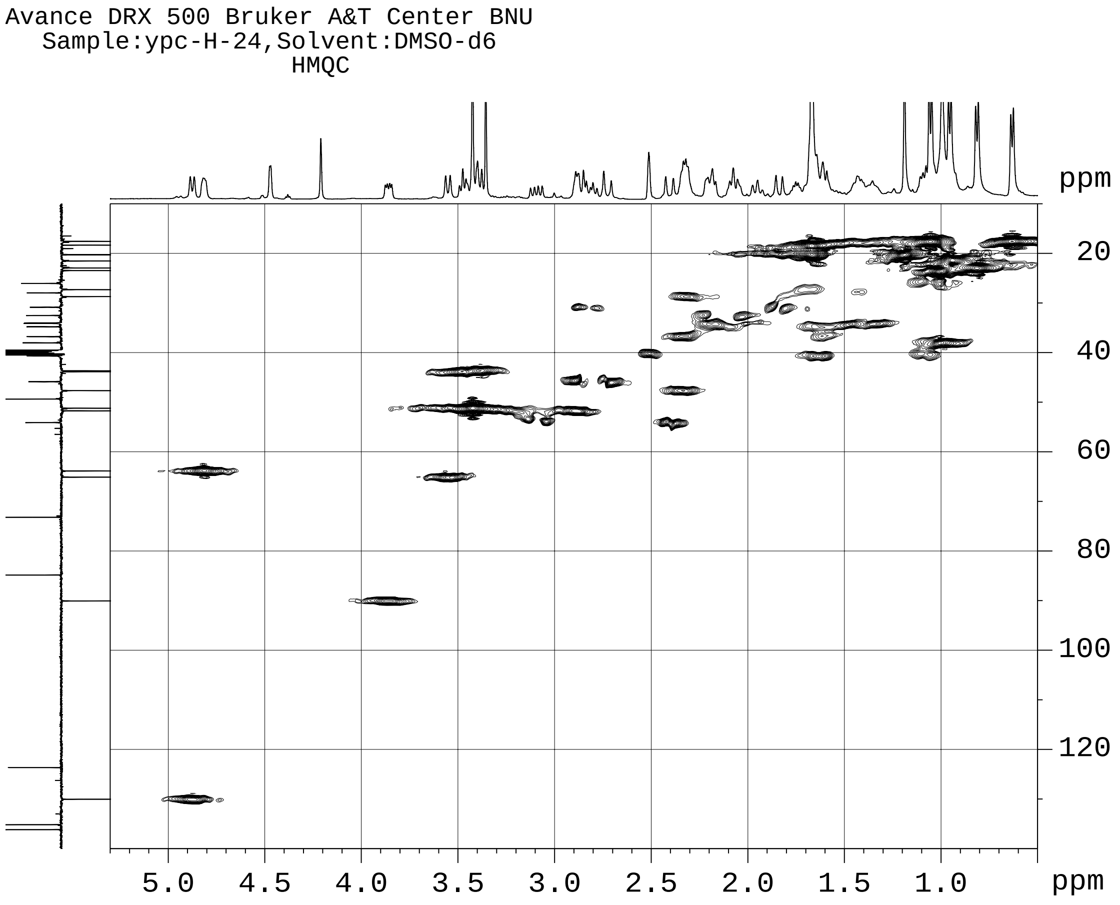

Figure S19 HMQC spectrum of compound **3**

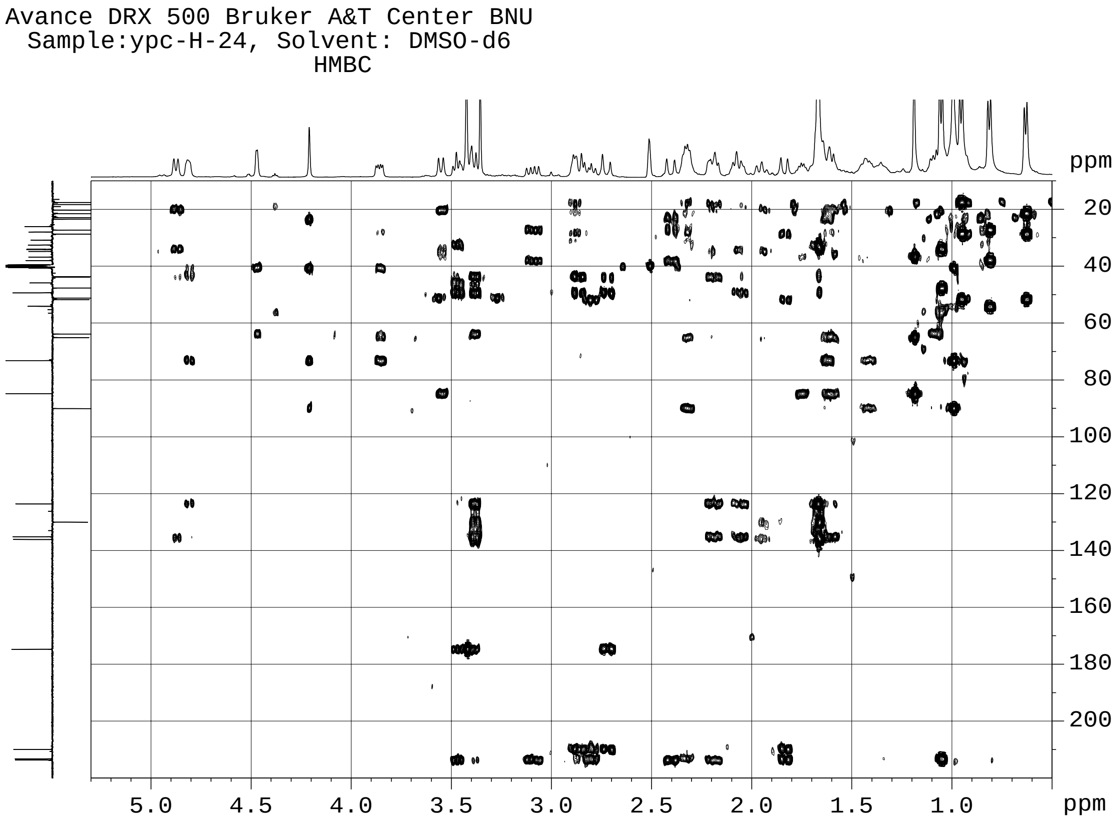

Figure S20 HMBC spectrum of compound **3**

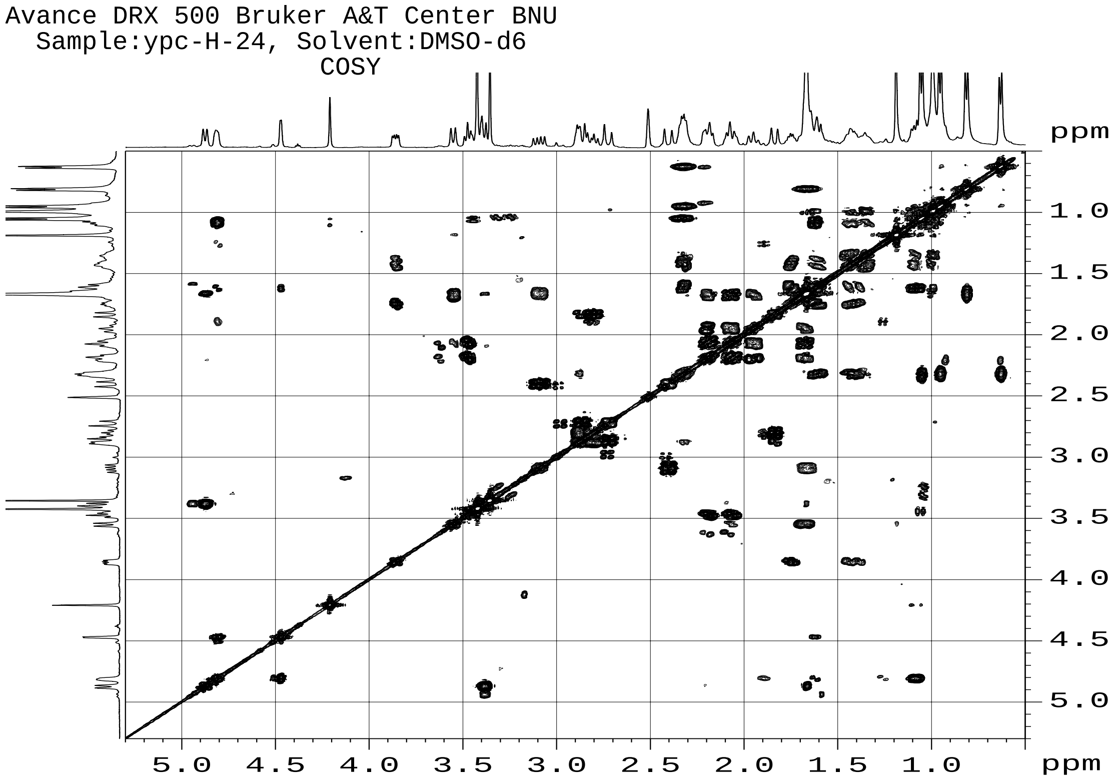

Figure S21 COSY spectrum of compound **3**

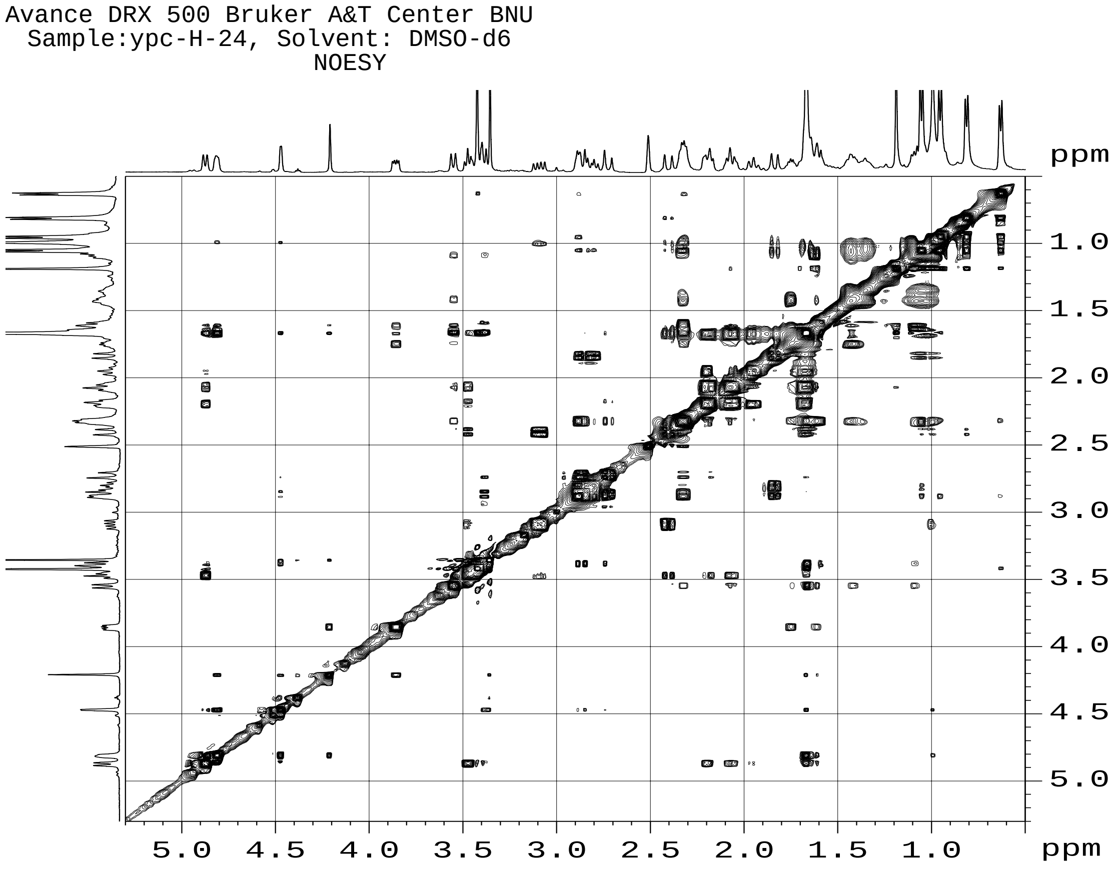

Figure S22 NOESY spectrum of compound **3**

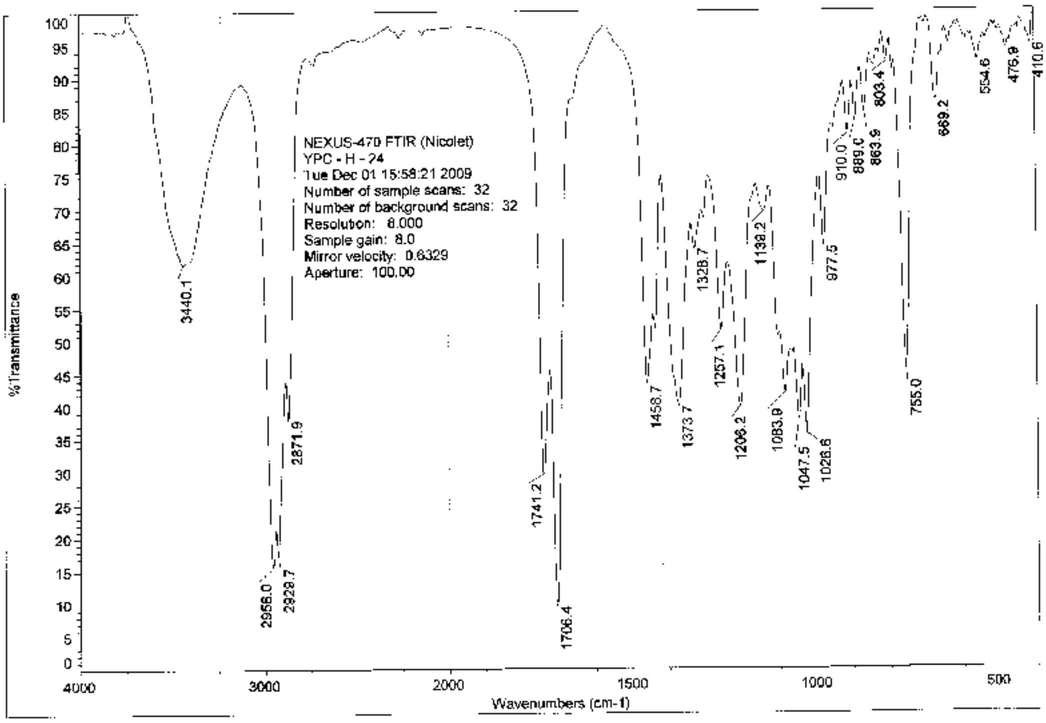

Figure S23 IR spectrum of compound **3**

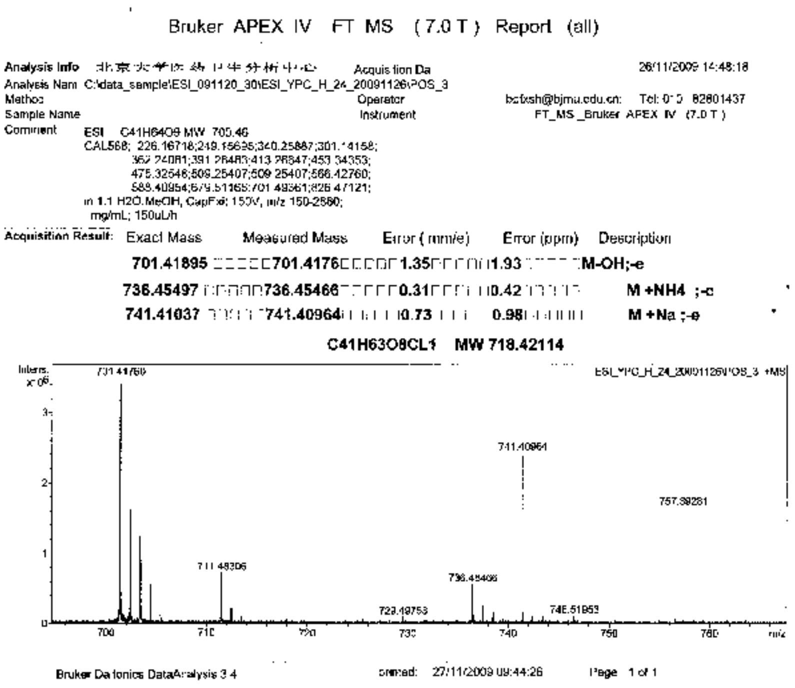

Figure S24 HRESIMS spectrum of compound **3**

Spectra of compound **4**

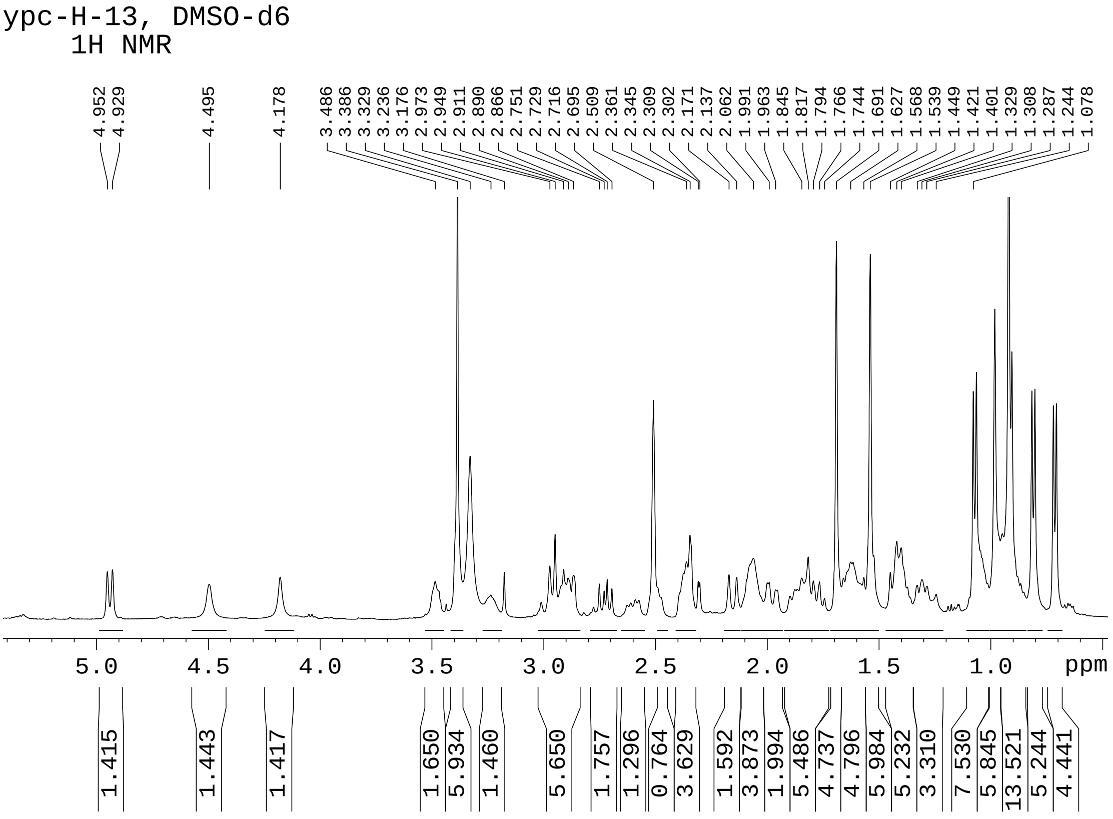

Figure S25 ^{1}H NMR spectrum of compound **4**

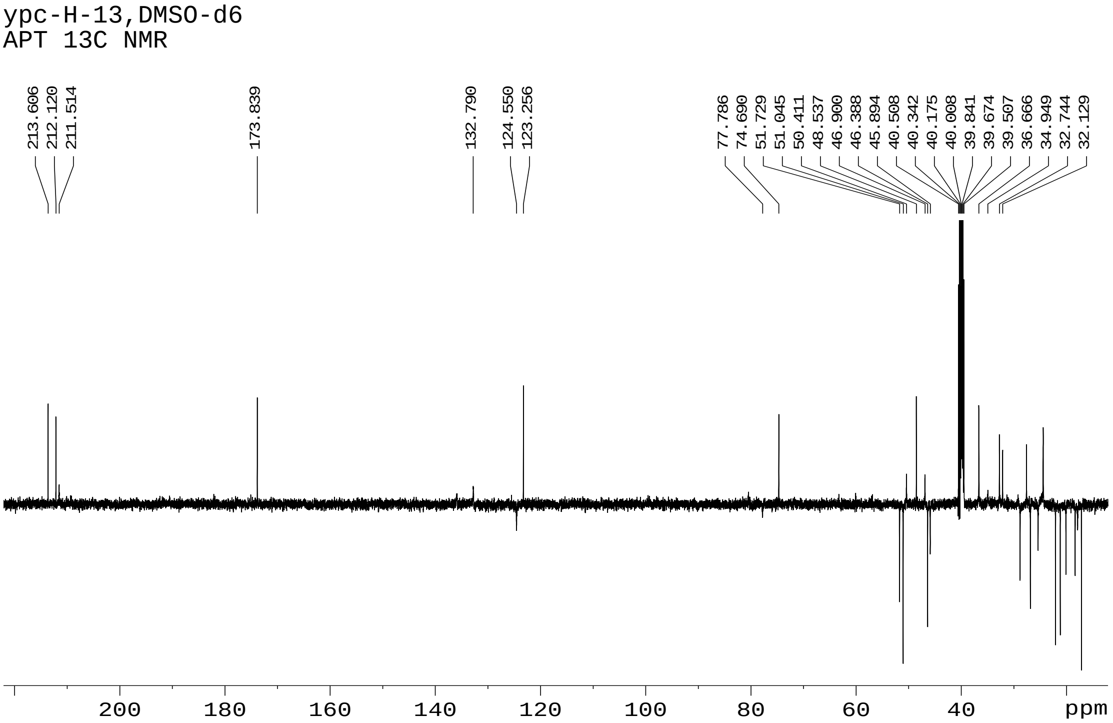

Figure S26 ^{13}CNMR spectrum of compound **4**

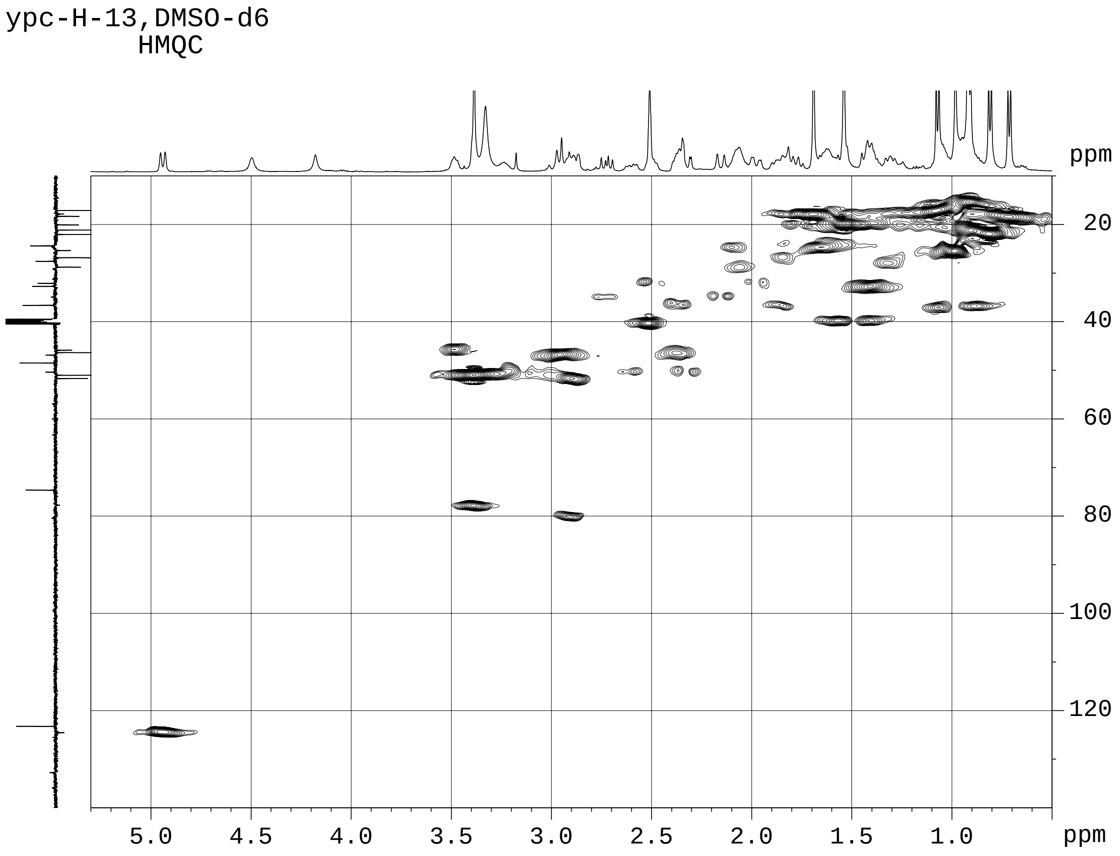

Figure S27 HMQC spectrum of compound **4**

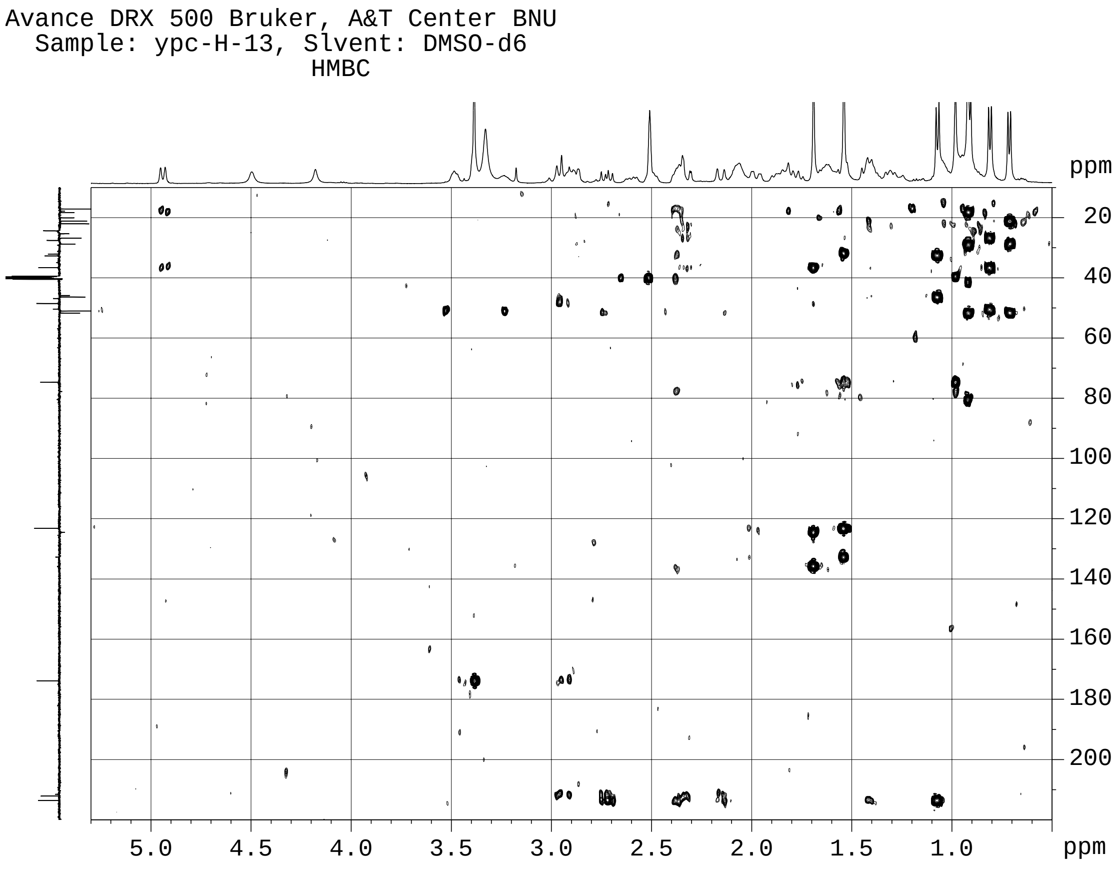

Figure S28 HMBC spectrum of compound **4**

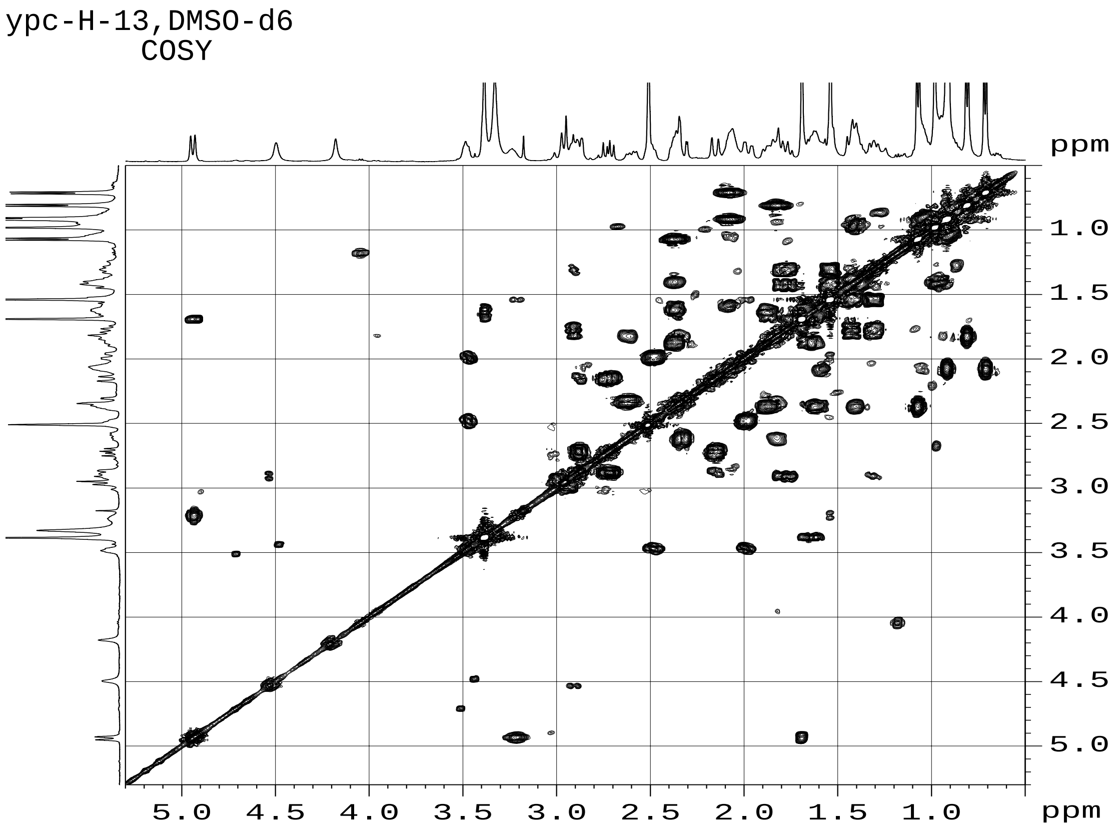

Figure S29 COSY spectrum of compound **4**

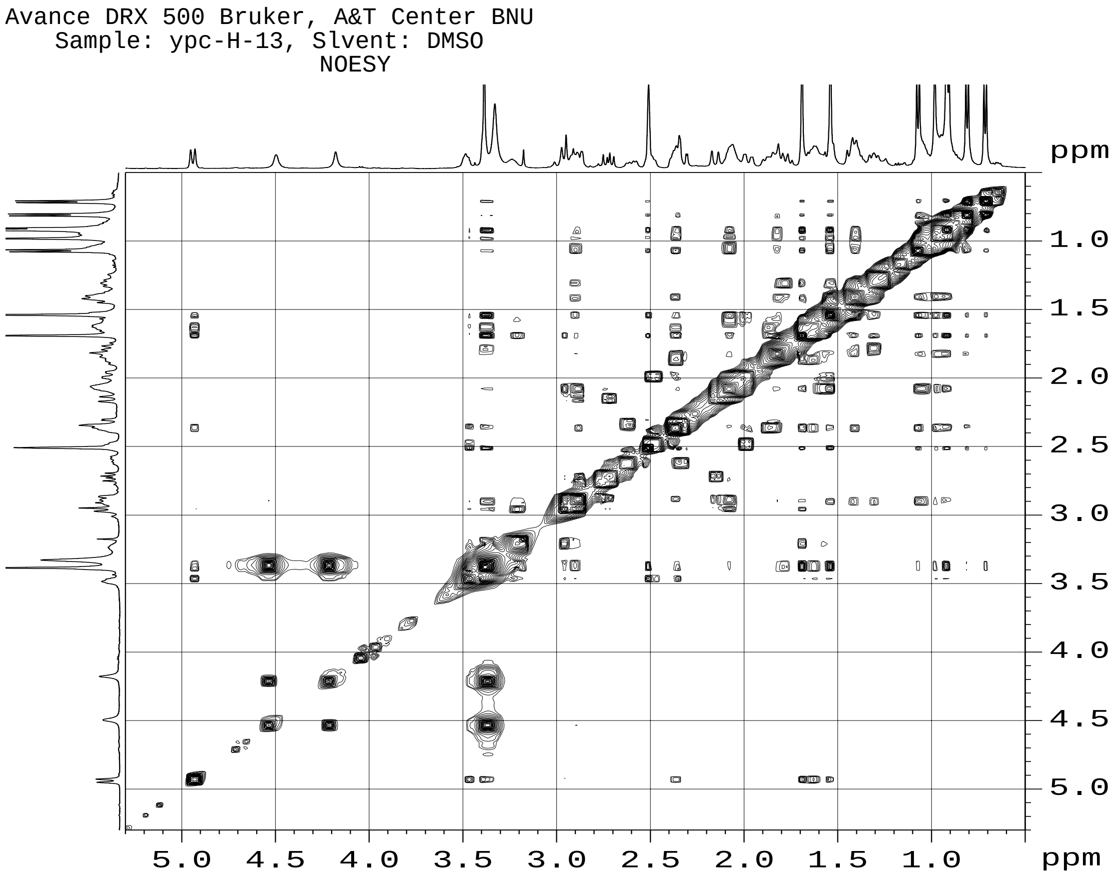

Figure S30 NOESY spectrum of compound **4**

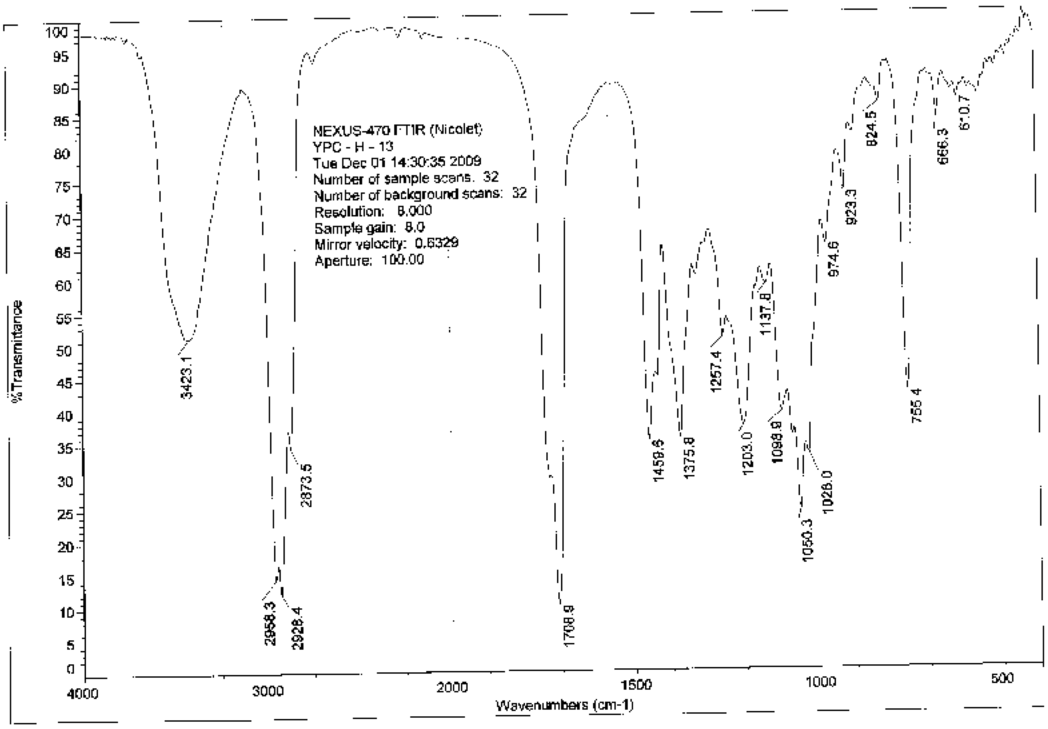

Figure S31 IR spectrum of compound **4**

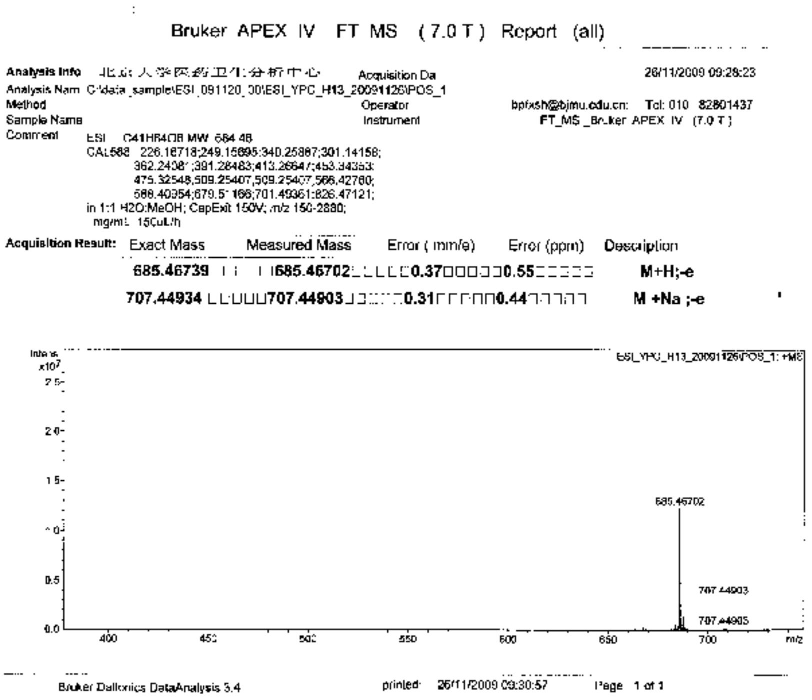

Figure S32 HRESIMS spectrum of compound **4**

Spectra of compound **5**

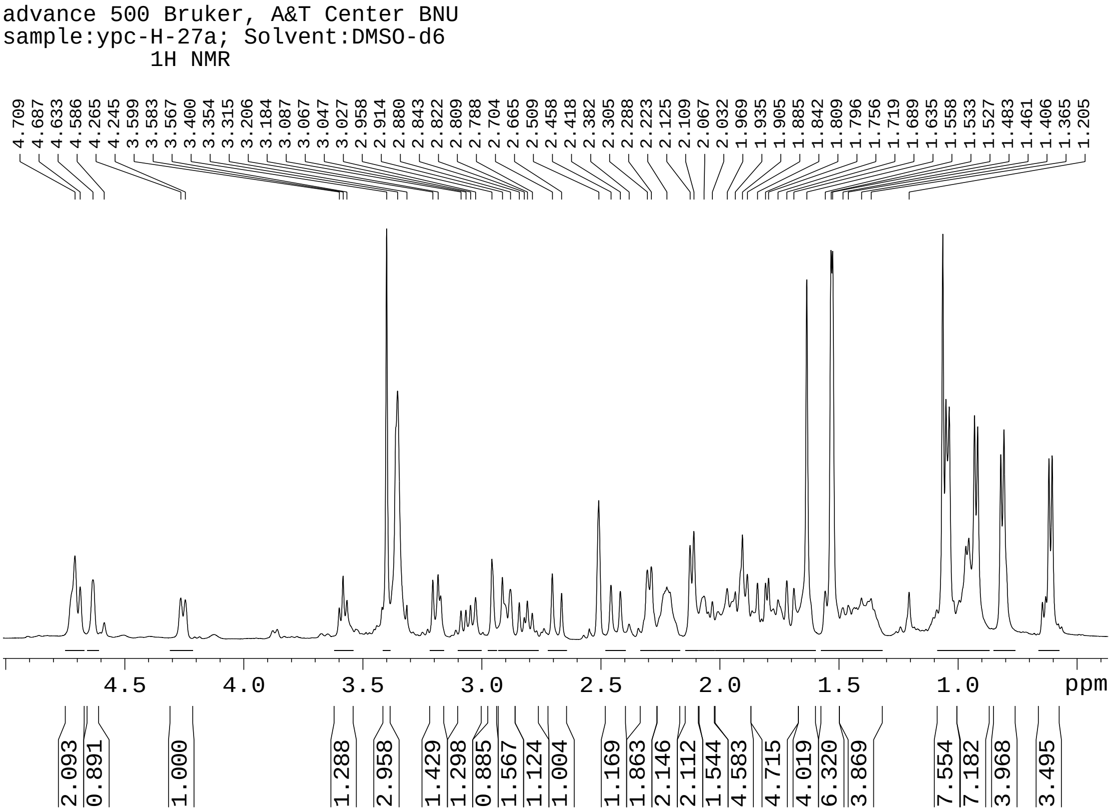

Figure S33 ^{1}H NMR spectrum of compound **5**

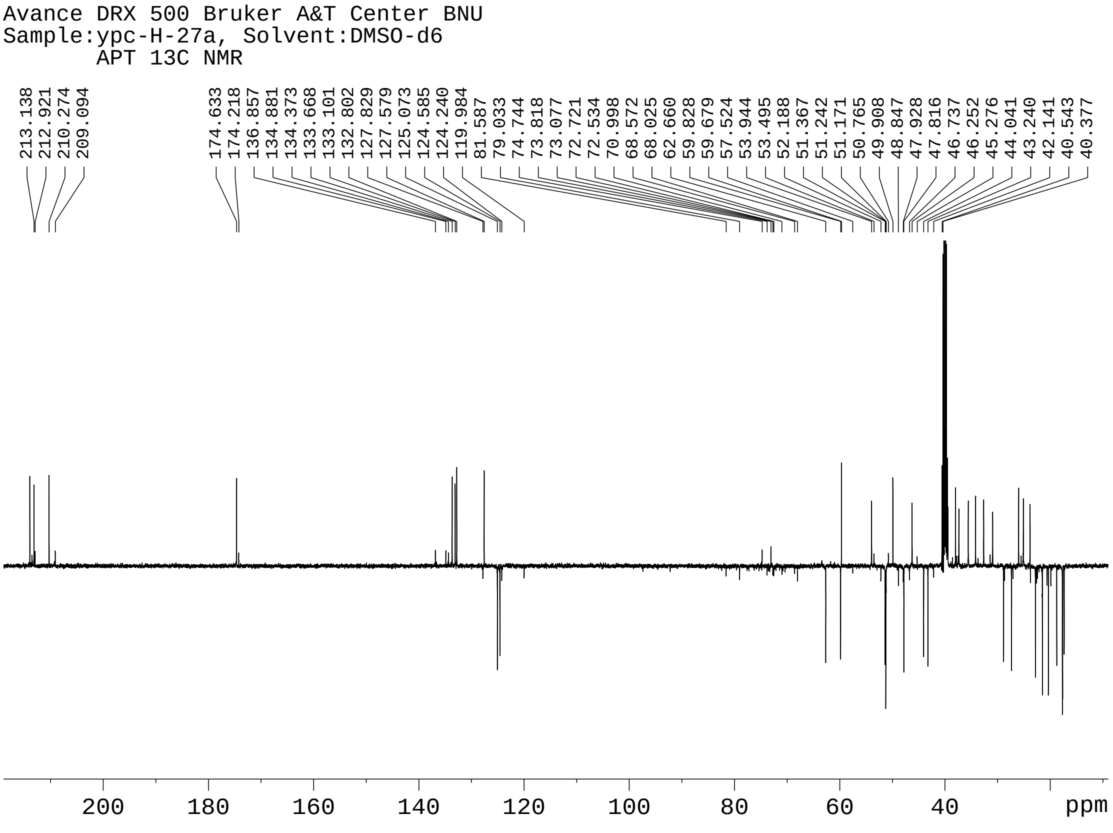

Figure S34 ^{13}C NMR spectrum of compound **5**

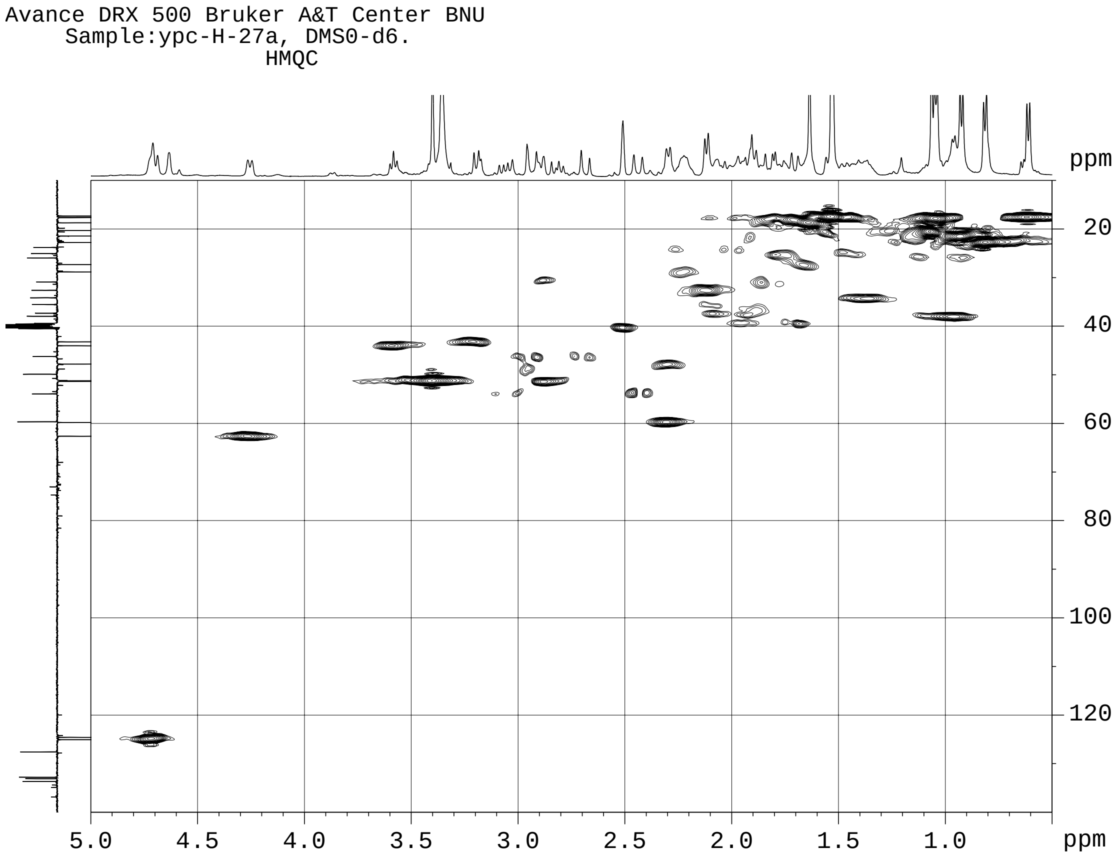

Figure S35 HMQC spectrum of compound **5**

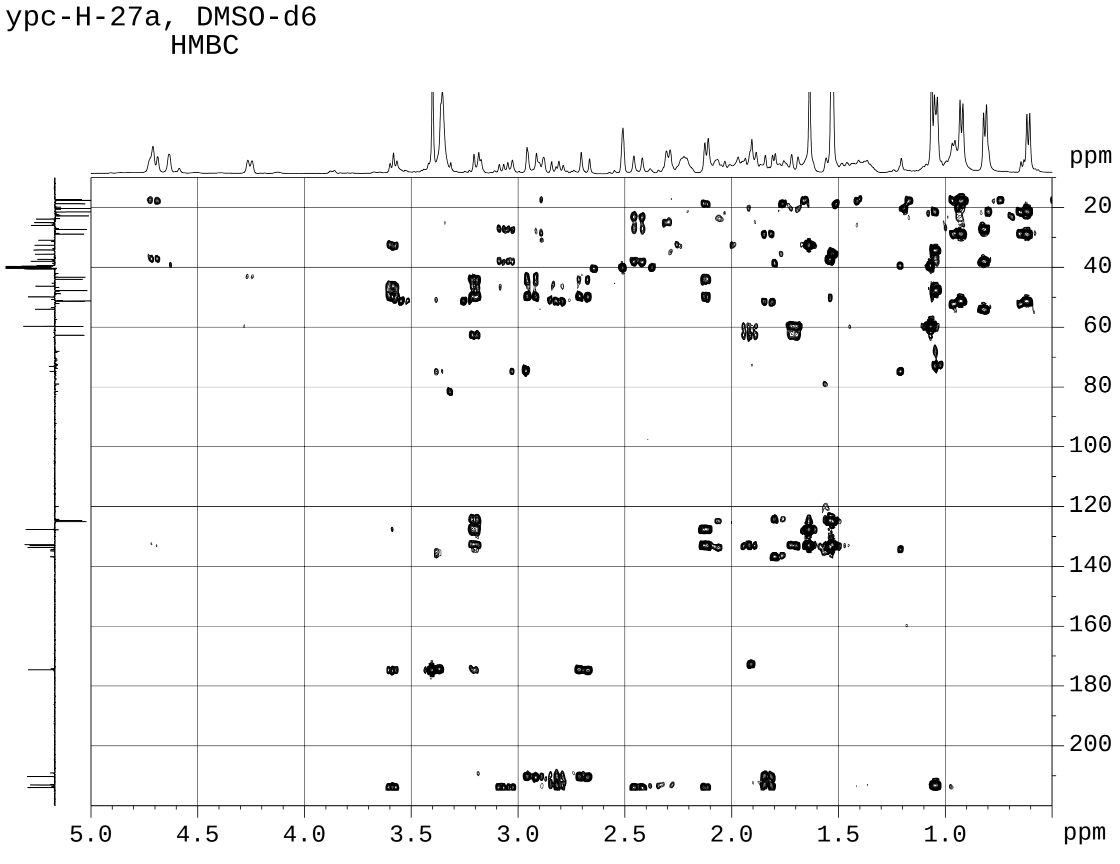

Figure S36 HMBC spectrum of compound **5**

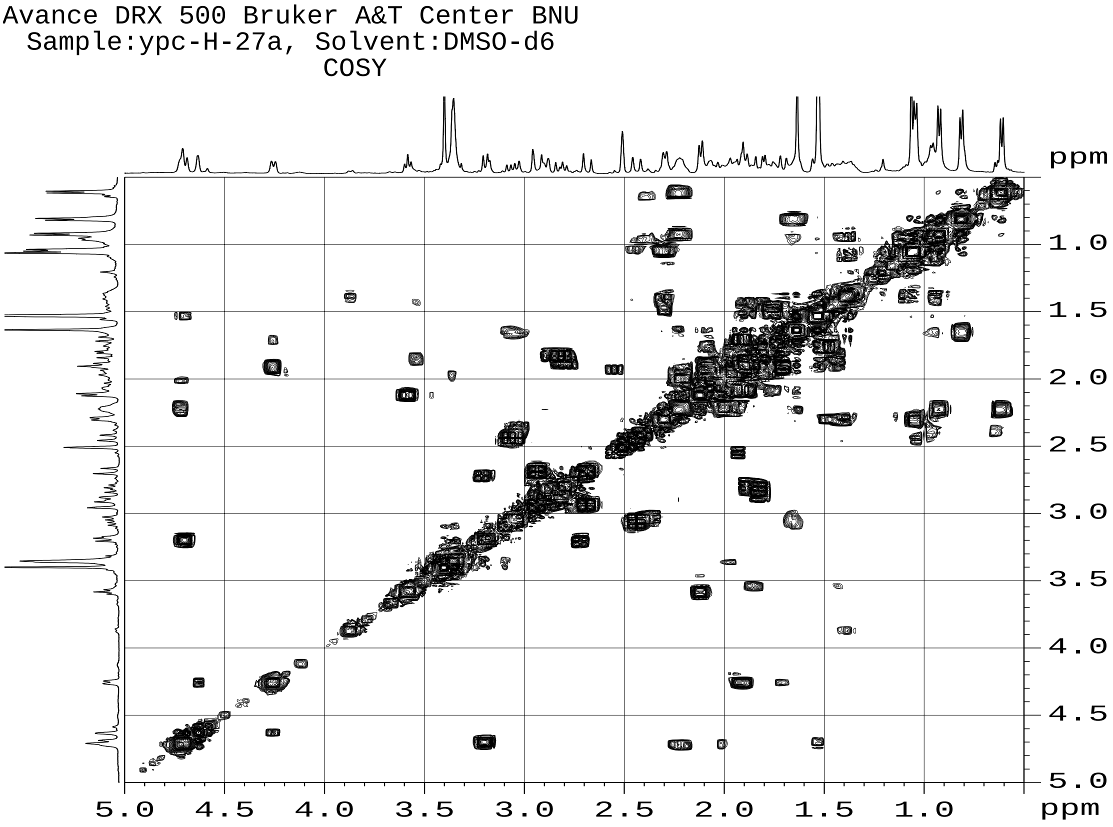

Figure S37 COSY spectrum of compound **5**

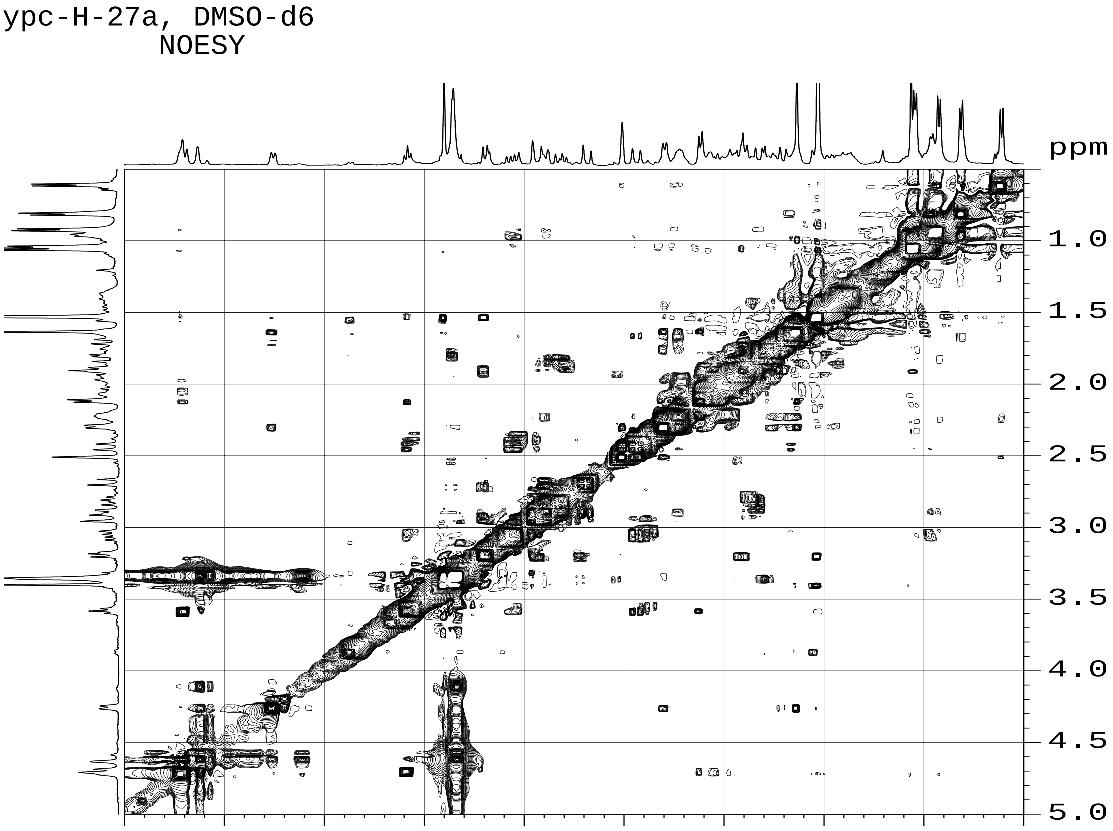

Figure S38 NOESY spectrum of compound **5**

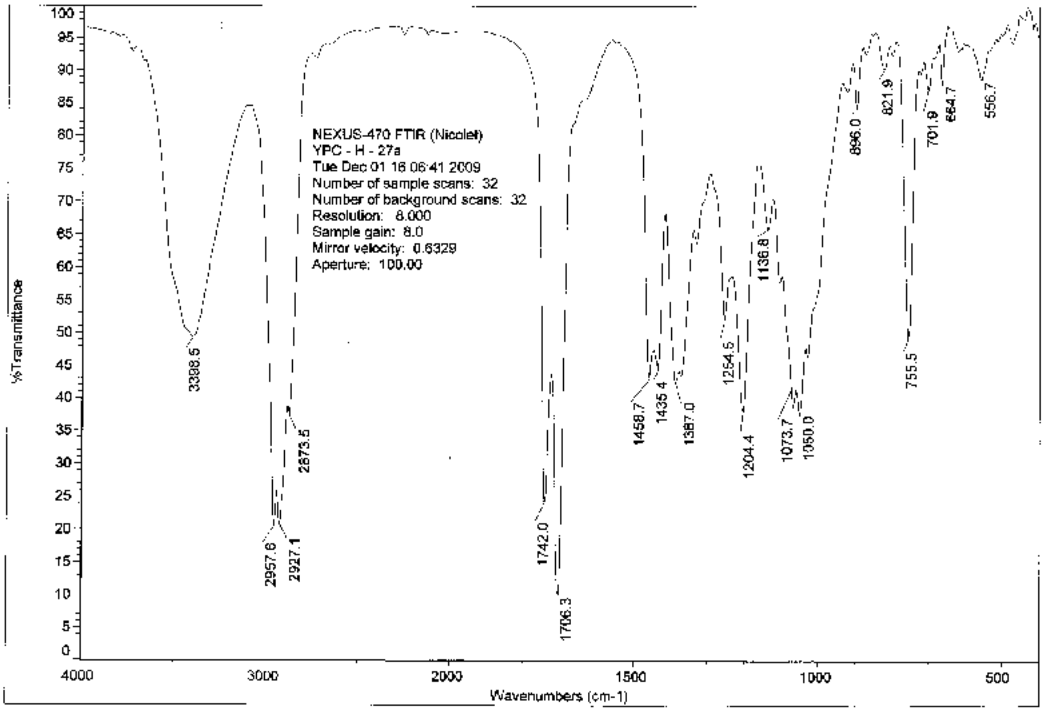

Figure S39 IR spectrum of compound **5**

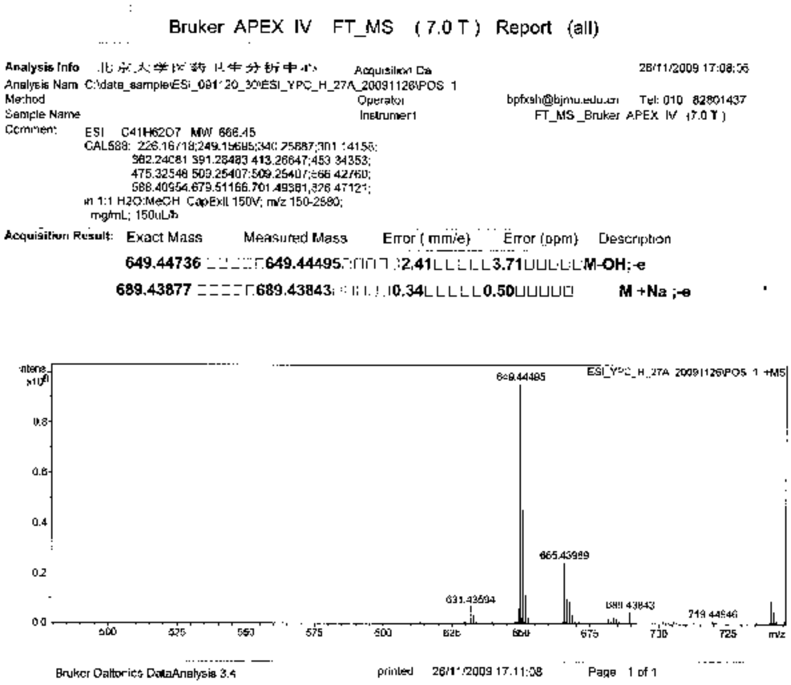

Figure S40 HRESIMS spectrum of compound **5**

Spectra of compound 6

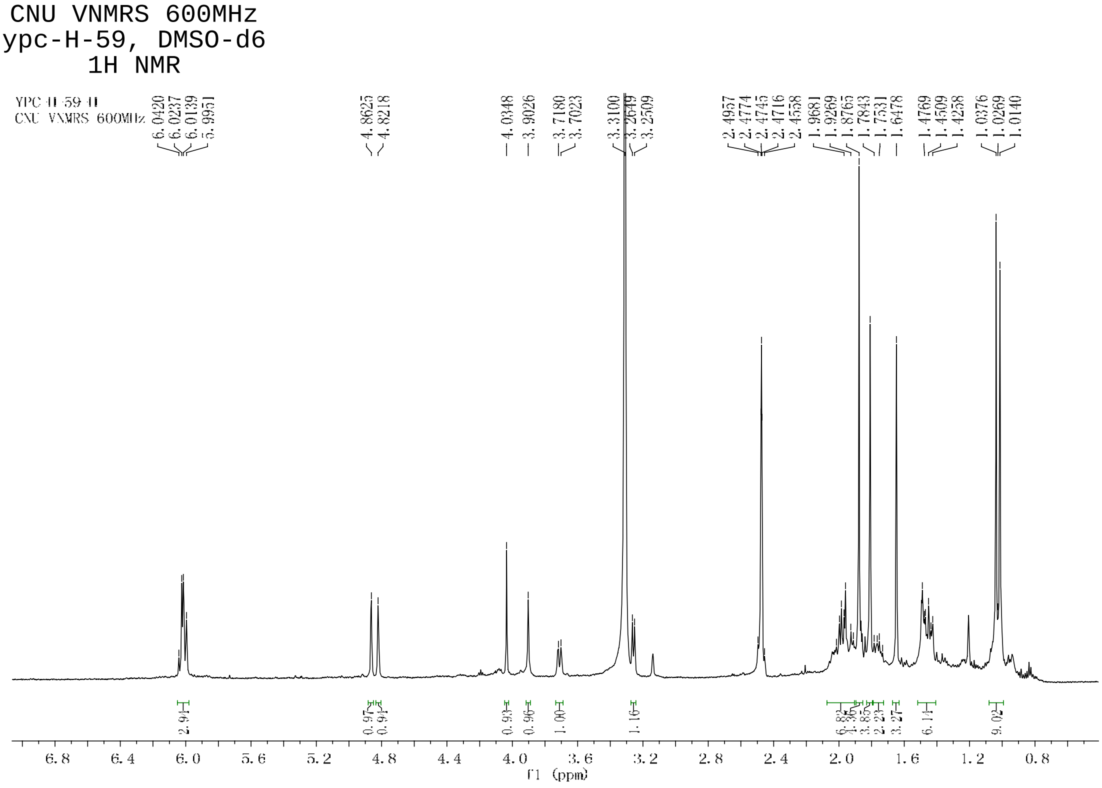

Figure S41 ^{1}H NMR spectrum of compound **6**

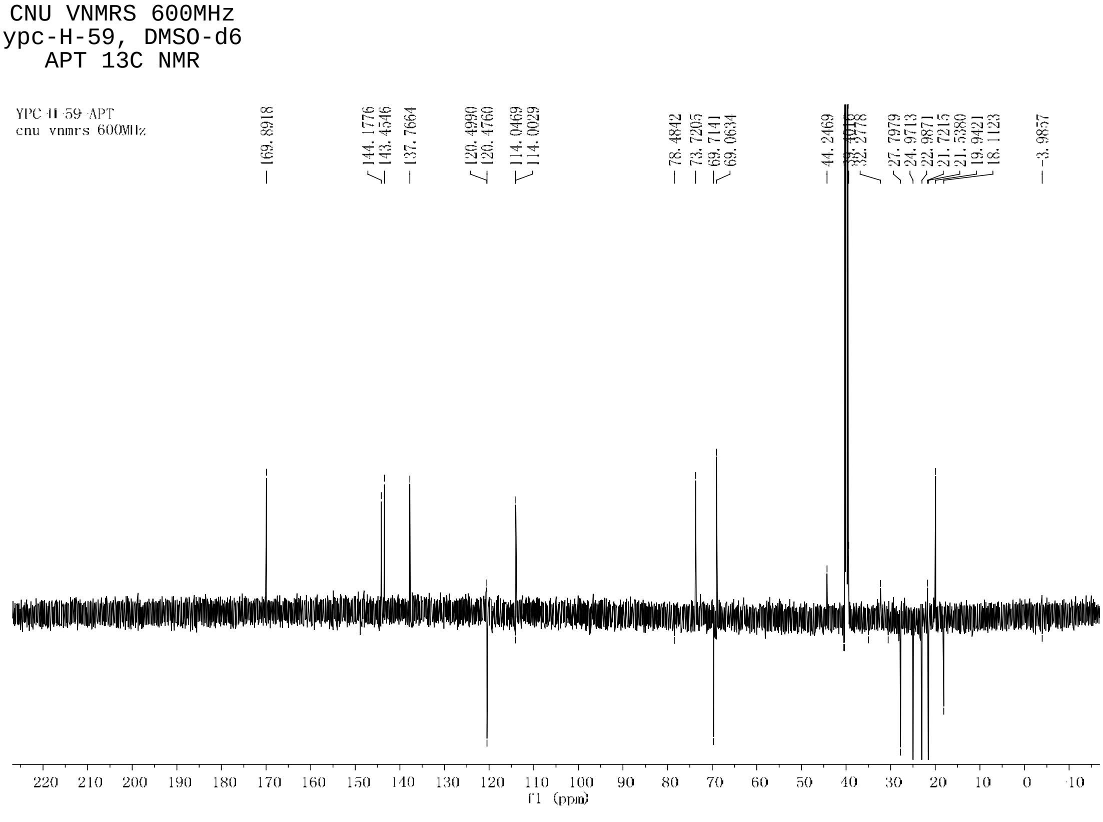

Figure S42 ^{13}C NMR spectrum of compound **6**

CNU VNMRS 600MHz
ypc-H-59, DMSO-d6
HSQC

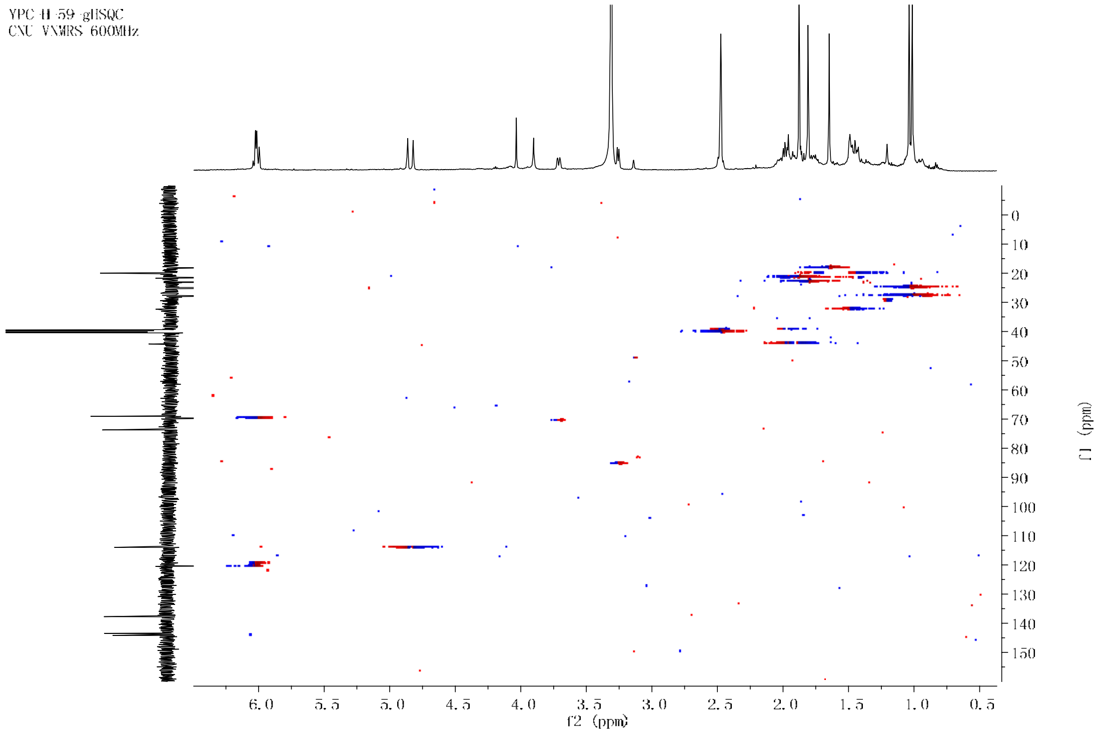

Figure S43 HSQC spectrum of compound **6**

CNU VNMRS 600MHz
ypc-H-59, DMSO-d6
HMBC

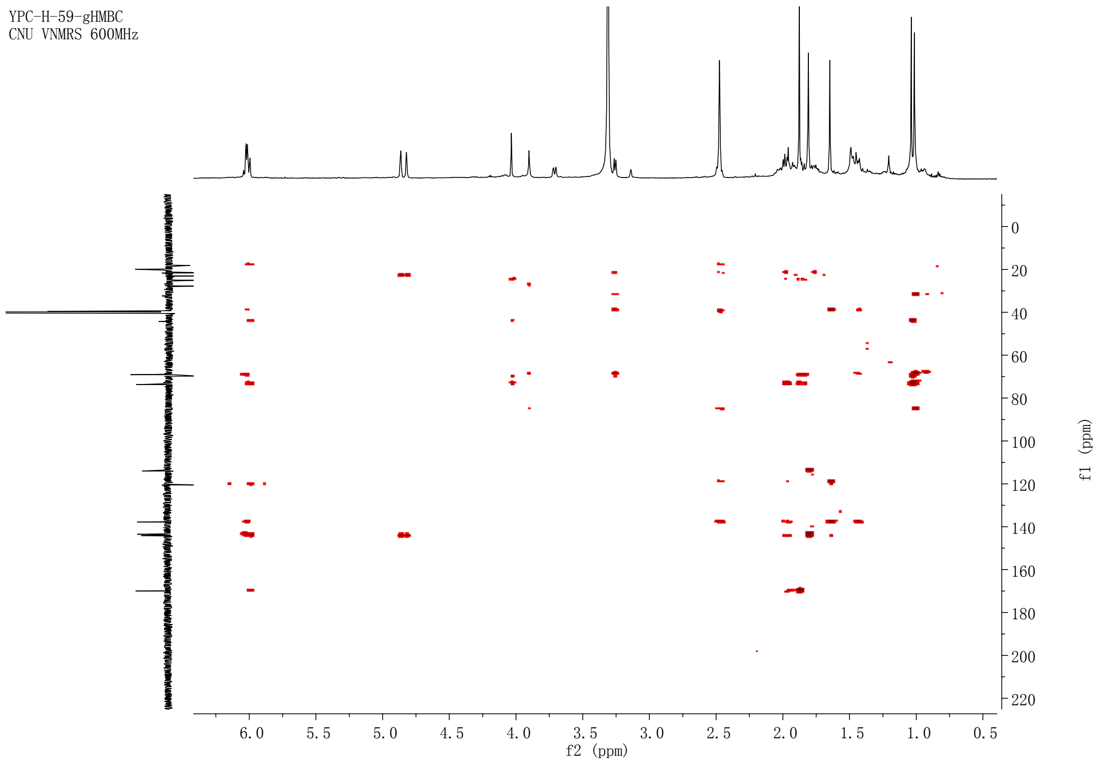

Figure S44 HMBC spectrum of compound **6**

CNU VNMRS 600MHz
ypc-H-59, DMSO-d6
COSY

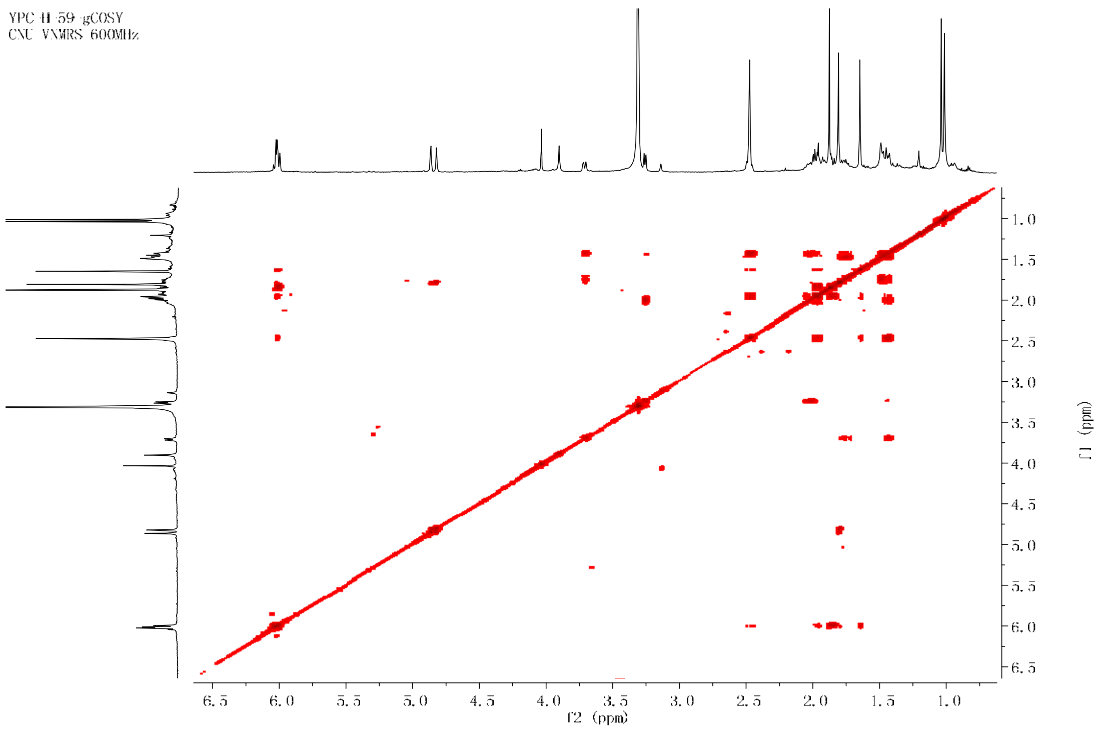

Figure S45 COSY spectrum of compound **6**

CNU VNMRS 600MHz
ypc-H-59, DMSO-d6
NOESY

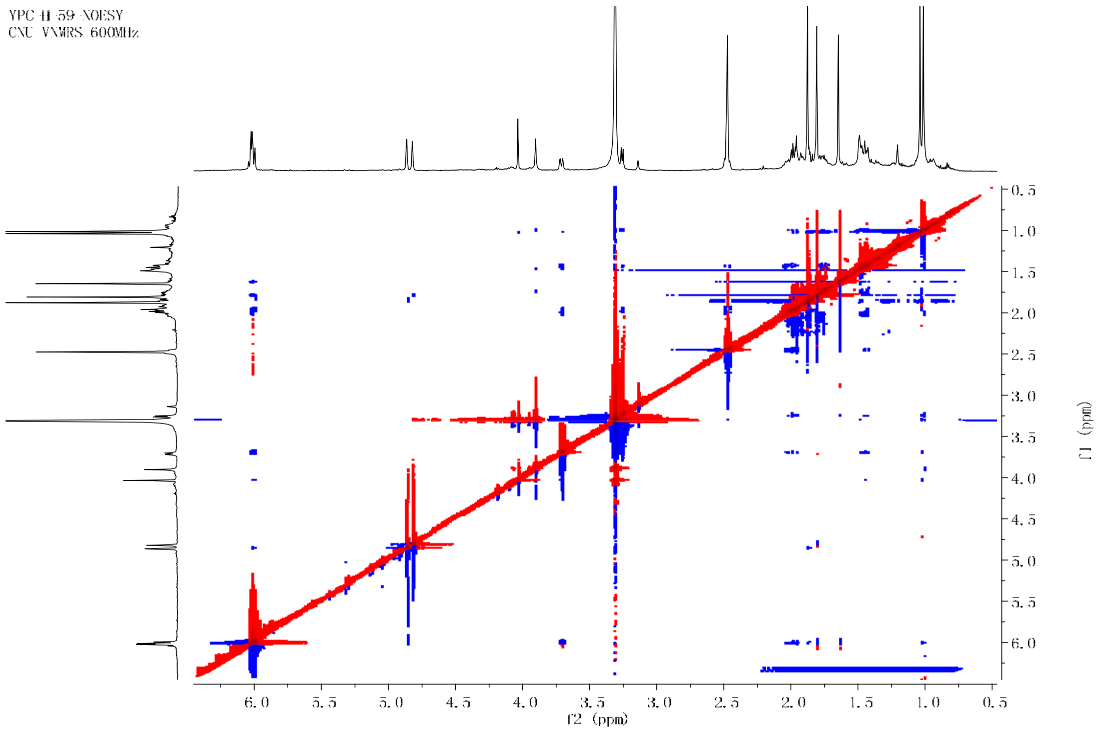

Figure S46 NOESY spectrum of compound **6**

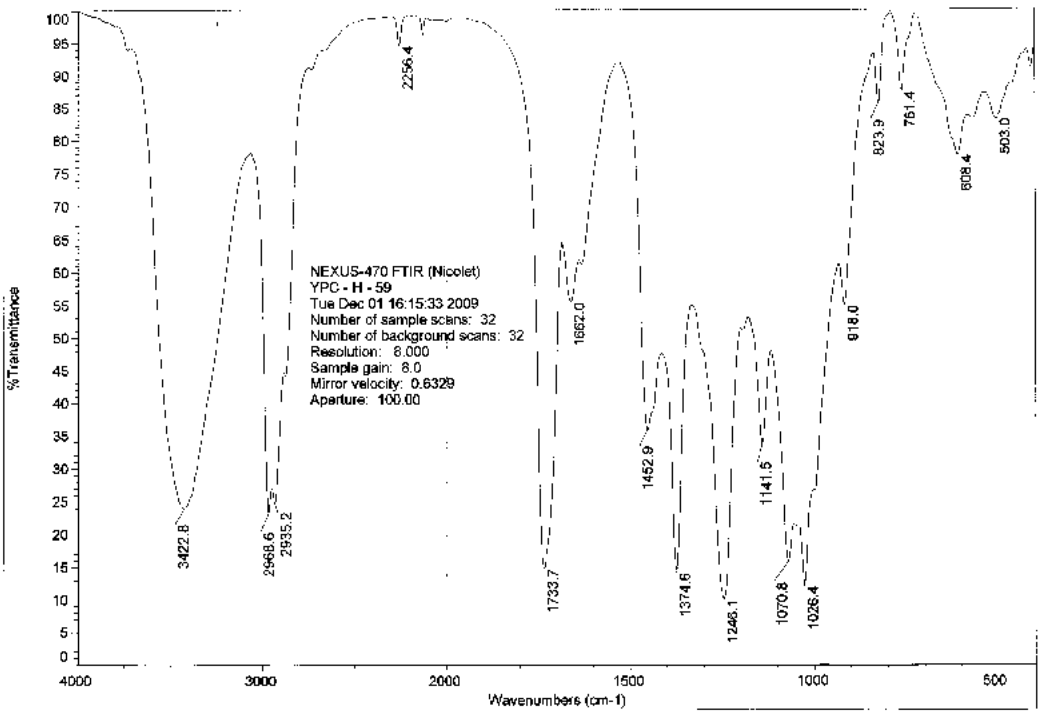

Figure S47 IR spectrum of compound **6**

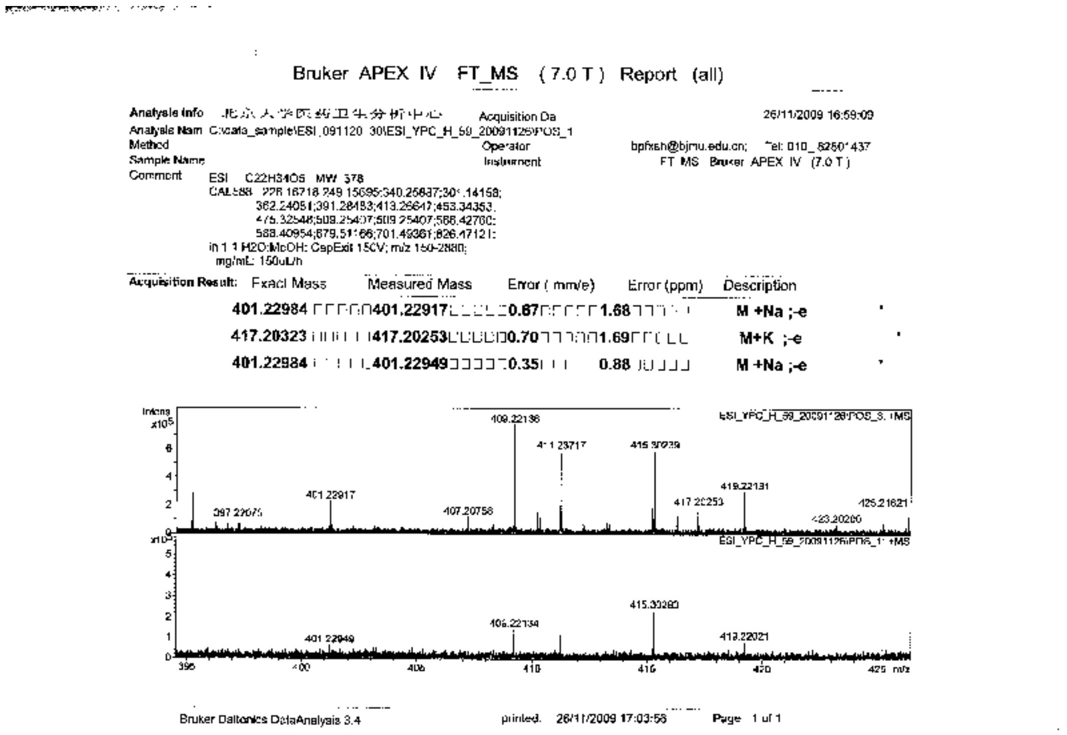

Figure S48 HRESIMS spectrum of compound **6**
